# Mobile health interventions to improve adherence to oral anticoagulant treatment: A systematic review and critical analysis

**DOI:** 10.1002/bcp.70718

**Published:** 2026-07-31

**Authors:** Non Davies, Elin Haf Davies, Dyfrig A. Hughes

**Affiliations:** ^1^ Centre for Health Economics and Medicines Evaluation, North Wales Medical School Bangor University Bangor UK; ^2^ Aparito, a wholly owned subsidiary of Eli Lilly and Company Wrexham UK

**Keywords:** medication adherence, mobile health, oral anticoagulants, randomized controlled trials

## Abstract

**Aims:**

Non‐adherence to oral anticoagulants (OACs) is associated with significant morbidity and mortality. Mobile health (mHealth) interventions have been used to improve medication adherence across the three phases of initiation, implementation and persistence. This review investigated the effectiveness of mHealth interventions in improving OAC adherence.

**Methods:**

PubMed, Embase, Cochrane Register and Web of Science were searched from 1/1/2000 to 14/11/2025 using terms identifying mHealth, OACs, medication adherence and randomized controlled trials. Included studies were assessed using the Risk of Bias for Interventional Adherence Studies tool. A narrative synthesis of the findings was undertaken. The study protocol was published in PROSPERO (CRD42022372863).

**Results:**

A total of 2571 studies were screened from which 24 randomized controlled trials met the criteria for inclusion. Adherence was most frequently assessed electronically using smart pill bottles and medication events monitoring systems. Nine of the 13 trials reporting statistically significant improvements in adherence tested smartphone‐based apps, telephone calls or text messages to support participants or as medication intake reminders. In terms of methodological quality, studies scored best with respect to randomization and blinding procedures, data analysis and interpretation. Studies scored worst in relation to study design and implementation of procedures and adherence outcome measurement and reporting.

**Conclusions:**

mHealth interventions involving smartphone‐based apps or telephone and text messages may be effective in improving adherence to OACs. Future research should focus on optimizing the frequency of delivery, content of calls or messages and automation. Research should prioritize larger, longer‐term trials with emphasis placed on trial design, conduct and reporting.

## 1 INTRODUCTION

Worldwide, vitamin K antagonists have been the mainstay oral anticoagulants (OACs) for stroke prevention and treatment of venous thrombosis for the last 60 years. More recently, however, there has been a shift towards the use of direct oral anticoagulants (DOACs), to the extent that they are now often the preferred choice—mainly due to the reduced need for monitoring and the associated improved convenience to patients, lower incidence of major bleeding and fewer food‐ and drug–drug interactions.^1^

Despite their several advantages, adherence to DOACs is a major problem, with real‐world evidence indicating that around 20% of patients discontinue treatment prematurely and 40% of patients do not take their doses as prescribed.^2^, ^3^, ^4^ Missed and delayed dosing, representing the implementation phase of adherence,^5^ is potentially more hazardous with DOACs than with warfarin due to their shorter durations of action.^2^

Non‐adherence to medication is a complex phenomenon, with several determinants often contributing. The World Health Organization (WHO) categorized these according to whether they are factors related to the patient, their socio‐economic circumstances, healthcare systems, condition or treatment.^6^ Barriers related to the patient may include inadequate motivation, a low educational level and beliefs about medicines. Barriers related to the treatment itself may include its complexity, cost and the adverse effects experienced.^7^ In the case of DOACs, less frequent contact with clinicians due to the reduced need for monitoring may represent missed opportunities for providing adherence support.^8^

One potential method for improving medication adherence involves mobile health (mHealth) technologies.^9^ mHealth is defined by the WHO as ‘medical and public health practice supported by mobile devices, such as mobile phones, patient monitoring devices, personal digital assistants, and other wireless devices’.^10^ Most mHealth interventions are based on smartphones due to their popularity.^9^ The COVID‐19 pandemic has been a prominent driver for mHealth development, with technologies that allow patients to be monitored in real time, in remote settings, proving invaluable in times when patient contact was restricted.^11^

There have been numerous studies of the effectiveness of mHealth adherence‐enhancing interventions for chronic disease management. For instance, a review by Xu and Long^12^ reported that app‐based interventions may be useful in hypertension management by promoting medication adherence. A review by Qi et al^13^ found that digital solutions can improve the adherence of patients with cardiovascular disease; while a meta‐analysis of clinical trials in chronic diseases showed a positive impact of mobile apps on improving medication adherence.^14^ In the context of DOACs, mHealth offers a potential substitute for the patient/clinician interaction that has occurred historically via anticoagulation clinics for the management of warfarin. The aim of this systematic review is to determine the effectiveness of mHealth interventions to promote adherence to OACs.

## 2 METHODS

The protocol for this review is registered with the International Prospective Register of Systematic Reviews (PROSPERO), registration number CRD42022372863.

### 2.1 Eligibility criteria

The review included studies whose participants were ≥18 years of age. Participants were required to be self‐responsible for taking OACs, defined as personally administering their medication without the direct involvement of caregivers or healthcare professionals. We did not exclude studies that used dose administration aids. OACs could be either vitamin K antagonists or DOACs. The review focused on randomized controlled trial (RCT) evidence, where patients were allocated to an mHealth intervention aimed at promoting medication adherence or a suitable control, such as routine care. Included studies were required to include a measure of medication adherence as an outcome and published in the English language.

### 2.2 Information sources

The databases PubMed, Embase, Cochrane Central Register of Controlled Trials (CENTRAL) and Web of Science were searched for articles published between 1/1/2000 and 14/11/2025.

### 2.3 Search strategy

Search terms were based on the concepts of (i) mHealth, (ii) OACs, (iii) medication adherence and (iv) RCTs. These terms were combined using Boolean ‘AND’ and ‘OR’ operators and not restricted by field, to increase the search sensitivity, as detailed in the Appendix S1.

### 2.4 Selection process

The search results were exported into the reference managing software, Zotero, and duplicate articles were removed manually. Study screening and selection was performed by one reviewer (N.D.), in line with the eligibility criteria. This was done initially by the titles and abstracts and then by the articles' full texts. The reviewer (N.D.) worked independently, with a second reviewer (D.H.) consulted in cases of uncertainty, to reach consensus. No automation tools were used during the selection process.

### 2.5 Data extraction

Data extraction was performed independently by one reviewer (N.D.). In the case of uncertainty, a second reviewer (D.H.) was consulted. No automation tools were used in the data collection process. When data were unclear or missing, study authors were contacted via email for clarification.

The following data were extracted: citation details, publication year, country, duration, sample size, demographics, study methodology, characteristics of the intervention and control groups, details of the outcomes assessed and their measures, study results and the authors' interpretation of the results.

### 2.6 Methodological quality (risk of bias assessment)

To assess the methodological quality of the studies included in the review, the risk of bias instrument for interventional adherence studies (RoBIAS) was employed.^15^, ^16^ This tool includes salient items from the International Society for Medication Adherence (ESPACOMP) Reporting Guideline (EMERGE)^17^ and the Timelines–Events–Objectives–Sources (TEOS) framework for medication adherence research.^18^, ^19^ Studies were assessed across four domains representing biases related to study design and implementation of study procedures, randomization and blinding procedures, adherence outcome measurement and reporting, data analysis and interpretation. These were rated using a scale of whether items were fully present (a score of 5) or fully absent (a score of 1). Items could also be rated as not being applicable (a score of 0). This produced an overall judgement for each domain, which collectively corresponded to an overall bias judgement ranging from low to critical.

### 2.7 Synthesis methods

Extracted data were tabulated, organized according to OAC and then by date. A meta‐analysis was indicated if studies were sufficiently homogeneous with respect to their methodology, intervention characteristics and reported outcomes. Alternatively, a narrative synthesis of the data would be undertaken in which studies would be grouped by the type of mHealth intervention employed, the adherence methods used and the outcomes assessed. In summarizing the findings, emphasis was placed on studies of highest methodological quality (lowest risk of bias) or largest in cohort size. The potential for intervention effects to be related to specific subgroups was considered based on the count of studies reporting statistically significant effects by OAC, dose frequency and disease/clinical indication.

### 2.8 Reporting

The reporting aimed to align with the synthesis without meta‐analysis (SWiM) and Preferred Reporting Items for Systematic reviews and Meta‐Analyses (PRISMA) guidelines.^20^, ^21^

### 2.9 Nomenclature of targets and ligands

Key protein targets and ligands in this article are hyperlinked to corresponding entries in https://www.guidetopharmacology.org/ and are permanently archived in the Concise Guide to PHARMACOLOGY 2025/26.^22^

## 3 RESULTS

A total of 2571 studies were identified. The removal of 661 duplicates resulted in 1910 potentially relevant articles. 1800 of these were excluded following title and abstract screening, resulting in 110 full‐text articles being sought for retrieval. All of these were obtained, of which 86 articles were excluded for the reasons detailed in Figure 1, leaving 24 studies^23^, ^24^, ^25^, ^26^, ^27^, ^28^, ^29^, ^30^, ^31^, ^32^, ^33^, ^34^, ^35^, ^36^, ^37^, ^38^, ^39^, ^40^, ^41^, ^42^, ^43^, ^44^, ^45^, ^46^ included in the final review.

**FIGURE 1:**
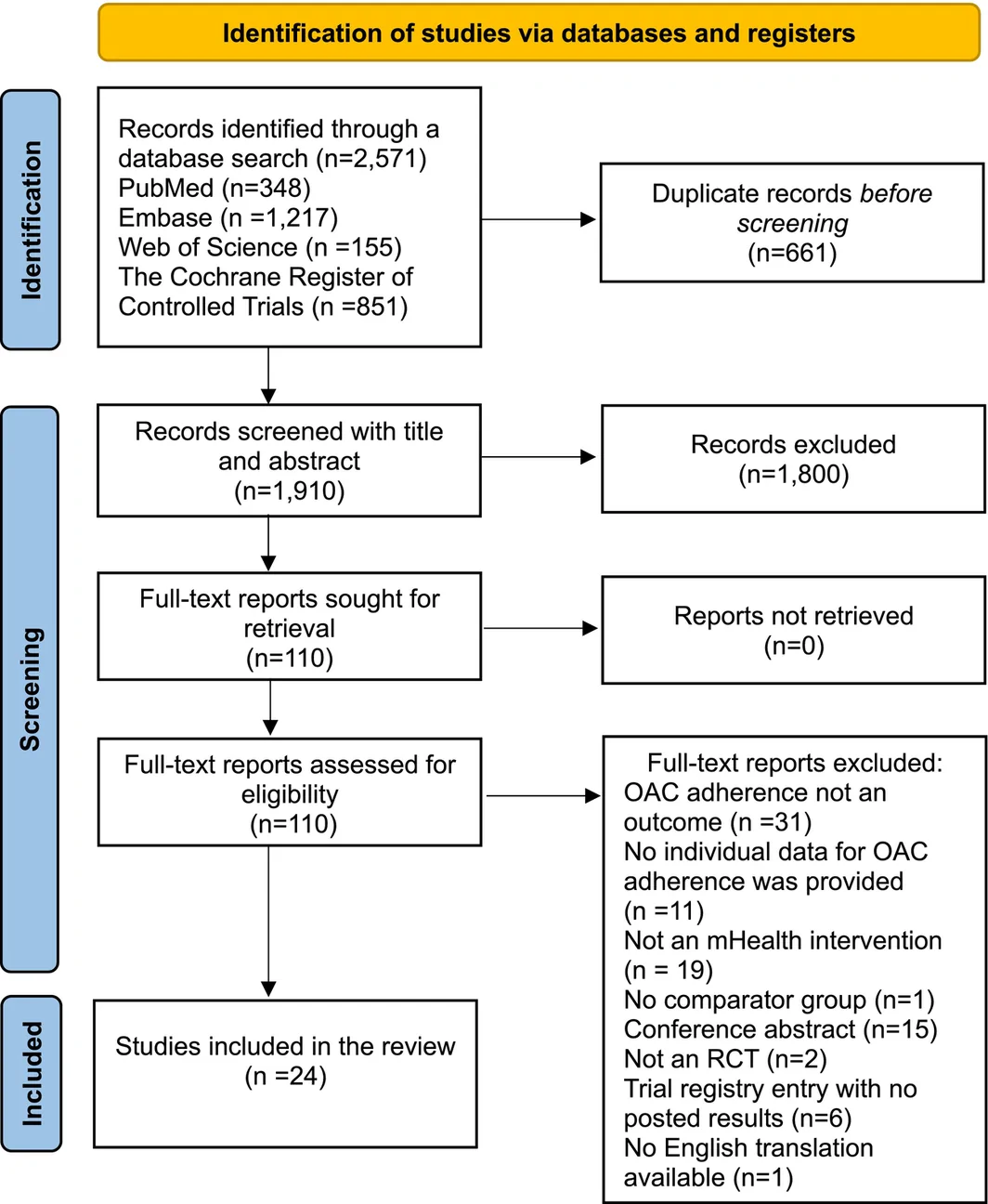
Preferred Reporting Items for Systematic Reviews and Meta‐analyses (PRISMA) flow diagram. Adapted from Page et al.^21^ mHealth, mobile health; OAC, oral anticoagulant; RCT, randomized controlled trial.

### 3.1 Study characteristics

Study characteristics are summarized in Table 1. A total of 11 150 participants were randomized across the 24 studies, recruited from 13 different countries. Most (11 studies) were conducted in the United States. Individual study sample sizes ranged from 28 to 3821, and follow‐up periods ranged from 30 days to 1 year. All studies were based in settings of ambulatory care, with no mean or median age of participants falling below 51 years. Overall, 44.8% of participants were female. The majority of RCTs recruited patients with non‐valvular atrial fibrillation with or without a prior history of ischaemic stroke or transient ischaemic attack; two studies included patients with venous thromboembolism.

**TABLE 1:** Characteristics of the included studies.

References	Study design	Inclusion criteria	Sample size	Sample demographics (age in years)	Follow‐up period
[23]	A parallel, randomized, controlled trial of lottery‐based incentives for improving warfarin anticoagulation control (investigators and analysts blinded)	Aged ≥21 years Receiving care at the University of Pennsylvania Anticoagulation Management Centre Target INR between 2 and 3 Have previously achieved stable anticoagulation with warfarin	Control = 48 Intervention = 53	Median age = 59.5 (control), 64.0 (intervention) 44% female	6 months
[24]	A multicentre, parallel, cluster‐randomized, controlled trial of an interactive website for improving adherence to warfarin, statins, antiplatelets, antihypertensives and antidiabetics (blinding process not reported explicitly)	Aged ≥18 Suffered a stroke due to cerebral infarction/transient ischaemic attack Admitted to hospital within 30 days of occurrence but stable Independent beforehand	Control = 2026 (24 clusters) Intervention = 1795 (23 clusters)	Mean age = 60.36 (SD 11.66) (control), 61.48 (SD 11.47) (intervention) 31.94% female	12 months
[25]	A four‐arm, multicentre, parallel, randomized, controlled trial of lottery‐based incentives and reminders for improving out‐of‐range INR values (only coordinators, investigators and analysts blinded)	A functioning telephone line Expected to be on warfarin therapy for a ≥6 months INR between 2.0 and 3.5 Experienced a ≥1 out‐of‐range INR reading within 90 days before study enrolment/at study enrolment	Control = 68 Interventions = 67 (lottery), 67 (reminder), 68 (lottery + reminder)	Overall median age = 62 32% female	6 months
[26]	A single‐blind randomized parallel‐group design study of the ‘banhu’ mobile app for patients who received mechanical heart valve replacement	Underwent mechanical heart valve replacement surgery under general anaesthesia Aged 18–65 Patients/family members can effectively use the smartphone app and are cooperative Education to at least a primary school grade Patients or their guardians provided informed consent	Control = 43 Intervention = 41	Mean age = 52.00 (SD 1.08) (control), 51.32 (SD 0.77) (intervention) 63.1% female	6 months
[27]	A parallel, randomized, controlled trial of an artificial intelligence platform for improving adherence to warfarin and DOACs (blinding process not reported explicitly)	Diagnosis of ischaemic stroke (with/without preceding transient ischaemic attack, with an NIHSS score between 1 and 20) Prescribed OAC	Control = 13 Intervention = 15	Mean age = 57 (SD 13.2) 53.6% female	12 weeks
[28]	A parallel, randomized, controlled trial of an online educational portal (primary outcome not reported explicitly, group allocation was unblinded)	Adult cardiology outpatient at Midwestern US hospital Non‐valvular AF diagnosis OAC prescription Access to internet/computer MyChart account/ready to register	Control = 80 Intervention = 80	Mean age = 71.1 (SD 8.5) 37.50% female	6 months
[29]	A multicentre, prospective, parallel, randomized, controlled, feasibility study of an electronic OAC decision support tool for improving HRQL (researcher conducting final assessments blinded).	Aged ≥18 years Electrocardiogram displaying AF	Control = 36 Intervention = 36	Mean age = 65 (SD 11) 44% female	3 months
[30]	A single‐blind parallel arm randomized controlled trial of a mobile health intervention for rural patients with AF	Aged ≥18 years Electronic health record documented diagnosis of AF Prescription of anticoagulation (primary indication of thromboembolic stroke prevention) Residence in a rural municipality Adequately speak English for study participation No plan to relocate within 12 months	Control = 135 Intervention = 135	Median age = 73.1 (IQR 67.5–78.6) 60.4% female	12 months
[31]	A prospective, single‐centre, parallel‐arm, randomized controlled trial of a mobile health intervention for patients with AF (blinding process not specified)	Age ≥21 years Electronic health record documented diagnosis of AF Prescription of an oral anticoagulant (with thromboembolic stroke prevention being the primary indication for treatment) CHA_2_DS_2_VASc score of ≥2 Adequately speak English for informed consent No expectation of relocation within 12 months of enrolment	Control = 120 Intervention = 123	Mean age = 70.8 (SD 9.8) 64.2% female	12 months
[32]	A double‐blind, parallel, randomized, controlled trial of follow‐up telephone calls conducted by a clinical pharmacist (primary outcome not reported explicitly)	DVT diagnosis Discharged in the acute phase following treatment Received warfarin/rivaroxaban following discharge Voluntary participation	Control = 64 Intervention = 61	Mean age = 61.89 (SD 11.52) (control), 63.80 (SD 12.09) (intervention) 50.4% female	6 months
[33]	A single‐blind, parallel, multicentre, encounter‐randomized, controlled trial of an online OAC shared decision‐making tool used during an in‐person encounter	Aged ≥18 Have non‐valvular AF CHA_2_DS_2_‐VASc score ≥1 (men)/≥2 (women) Can read and comprehend the consent form	Control = 459 Intervention = 463	Mean age = 71 (SD 10) (control), 71 (SD 11) (intervention) 39.37% female	10 months
[34]	An open‐label, parallel, randomized, controlled trial of using electronic personal health records for health‐related education and management (primary outcome not reported explicitly)	Aged ≥18 years Diagnosed with AF Prescribed dabigatran for stroke prevention Can read and comprehend English Have Internet access	Control = 44 Intervention = 46	Mean age = 66 30% female	30 days
[35]	A single‐blind, three‐phase crossover, controlled trial, in which patients on DOACs were randomized to daily telemonitoring or telemonitoring with immediate telephone feedback in case of intake errors, followed by an observation phase without daily transmissions	Have consecutive AF Currently taking/starting apixaban or rivaroxaban	24 participants randomized were to each group	Mean age = 72 (SD 9) 50% female	9 months
[36]	A double‐blind, parallel, randomized, controlled trial of an educational programme for improving adherence to apixaban	Non‐valvular AF/atrial flutter diagnosis (new or existing) CHADS_2_ score ≥1	Control = 583 Intervention = 579	Mean age = 72.6 (SD 8.9) (control), 73.1 (SD 9.1) (intervention) 48.02% female	48 weeks
[37]	An open‐label, randomized, controlled trial of a blended digital and human intervention to improve DOAC adherence	Aged ≥18 Recently (≤90 days) prescribed rivaroxaban for AF Nonadherence risk of at least 1 of 4 Possess a smartphone	Control = 67 Intervention = 72	Mean age = 65 (SD 9.6) 30% female.	6 months
[38]	A cluster‐randomized (stratified by pharmacy size), controlled trial of telephone follow‐up calls paired with smartphone‐based reminders to improve adherence to dabigatran (blinding process not reported explicitly)	Aged ≥18 years Diagnosed with venous thromboembolism/non‐valvular AF Prescribed dabigatran for the first time Could be contacted by telephone Spoke Polish sufficiently for assessment	Control = 172 Intervention = 153	Mean age = 67.1 (SD 10.1) (control), 67.6 (SD 10.5) (intervention) 49.8% female	90 days
[39]	A parallel, randomized, controlled trial of a smartphone‐based application, paired with an online portal, for improving adherence to apixaban (blinding process not reported explicitly)	Have non‐valvular AF CHA_2_DS_2_VASc score of ≥2 Can be treated with apixaban for 6 months	Control = 50 Intervention = 50	Median age = 70.8 50% female	6 months
[40]	A randomized, crossover study of medication intake reminders for OAC adherence in stroke patients (blinding process not specified)	Hospitalized in the University Hospital of Basel for ischaemic stroke AF diagnosis Prescribed a DOAC and ≥2 other medicines Self‐administered their medication	Control = 44 Intervention = 44	Mean age = 76.5 (SD 9.1) 39% female	12 months
[41]	A prospective randomized controlled study of a behaviour‐change programme and nudge strategies for OAC adherence (blinding process not specified)	Non‐valvular atrial fibrillation diagnosis Aged ≥18 years Took novel oral anticoagulants (dabigatran or rivaroxaban) CHA_2_DS_2_‐VASc score of ≥1 Available by mobile phone after discharge Agreed to participate in this study and sign the informed consent.	Control = 58 Intervention = 72	Mean age = 63.69 (SD 5.82) (control), 65.68 (SD 8.87) (intervention) 44.2% female	6 months
[42]	An open‐label, multicentre randomized controlled trial of an app to improve adherence to edoxaban	Aged ≥19 years requiring oral anticoagulation therapy (CHA_2_DS_2_‐VASc score ≥1 for men and ≥2 for women) Already taking or newly prescribed edoxaban Able to use Android smartphones Fluent in Korean Voluntarily consent to participate	Control = 250 Intervention = 248	Mean age = 65.7 31.7% female	6 months
[43]	An open‐label, parallel, randomized, controlled trial of a smartphone‐based application to improve HRQL	Aged ≥18 years Have chronic AF Prescribed OAC for stroke prevention Speak adequate English to use the intervention	Control = 59 Intervention = 61	Mean age = 72.1 (SD 9.10) 51.7% female	30 days
[44]	A prospective, parallel, randomized, controlled trial of motivational interviewing to improve adherence to an unspecified OAC (participants and study personnel unblinded, but outcome and data analysist blinded)	Aged ≥18 AF diagnosis OAC prescription at discharge	Control = 509 Intervention = 500	Median age = 76.0 (control), 75.5 (intervention) 46.48% female	12 months
[45]	A multicentre randomized controlled trial of the ‘Alfalfa App’ in the management of oral anticoagulation in patients with atrial fibrillation (blinding process not specified)	Aged > 18 years AF diagnosed with electrocardiogram and 24‐h Holter	Control = 56 Intervention = 57	Mean age = 61.65 (SD 11.01) 38.5% female	12 months
[46]	An open label, prospective, multicentre, randomized clinical trial of targeted patient education on unplanned cardiovascular outcomes (with blinded endpoint adjudication)	Aged >18 AF or atrial flutter diagnosed with an electrocardiogram	Control = 346 Intervention = 345 (in‐person education), 347 (online education), 194 (app‐based education)	Mean age = 69.8 (SD 9.2) 30.7% female	18 months

Abbreviations: AF, atrial fibrillation; CHA_2_DS_2_‐VASc, congestive heart failure, hypertension, age ≥75 years, diabetes, stroke/transient ischaemic attack, thromboembolism, vascular disease, age 65–74 years, sex category; DOAC, direct oral anticoagulant; HRQL; health‐related quality of life; INR, international normalized ratio; MEMS, medical events monitoring system; MMAS‐8, 8‐part Morisky Medication Adherence Survey; NIHSS, NIH Stroke Scale, OAC, oral anticoagulation; PDC, proportion of days covered; TTR, time in therapeutic range.

### 3.2 Study methods

Details of the experimental methods are summarized in Table 2. The studies assessed both clinical and non‐clinical outcomes. Medication adherence was the primary outcome in 13 studies^24^, ^26^, ^27^, ^30^, ^31^, ^36^, ^37^, ^38^, ^39^, ^40^, ^42^, ^44^, ^45^ and reported as a secondary outcome in the other 11 studies.^23^, ^25^, ^28^, ^29^, ^32^, ^33^, ^34^, ^35^, ^41^, ^43^, ^46^ Five studies^23^, ^25^, ^29^, ^43^, ^46^ stated a primary outcome other than medication adherence, with two based on health‐related quality of life,^8^, ^43^ two based on anticoagulation control according to the international normalized ratio range^21^, ^24^ and one based on cardiovascular event occurrence.^46^ Five studies^28^, ^32^, ^34^, ^35^, ^41^ did not state the primary outcome explicitly, while the primary outcome results of one study^33^ were reported in a separate publication. Seven studies^27^, ^28^, ^29^, ^30^, ^31^, ^32^, ^33^ considered adherence to both warfarin and at least one DOAC, four studies^23^, ^24^, ^25^, ^26^ assessed adherence to warfarin alone, nine to DOACs,^34^, ^35^, ^36^, ^37^, ^38^, ^39^, ^40^, ^41^, ^42^ while it was not specified in four further studies.^43^, ^44^, ^45^, ^46^ Most RCTs used a parallel design, with two using a crossover design.^35^, ^40^ The reporting of the blinding processes used was generally poor, with eight studies not reporting their blinding process explicitly^24^, ^27^, ^31^, ^38^, ^39^, ^40^, ^41^, ^45^ and just two studies reporting double‐blind allocation.^32^, ^36^ The sample size calculation was not reported in six studies.^26^, ^27^, ^32^, ^34^, ^35^, ^38^

**TABLE 2:** Study methods.

References	Intervention	Comparator	Other non‐mhealth aspects of the study	Primary outcome and measure(s)	Secondary outcome(s) and measure(s)
[23]	A daily lottery, administered via the Med‐emonitor. Upon opening the monitor and confirming warfarin intake, participants had a chance of winning $10 (one in five) or $100 (one in 100) daily	Usual clinic visits at 2 weeks and 3 and 6 months	No additional interventions were given during usual clinic visits	1 Warfarin anticoagulation control (percentage of out‐of‐range INR)	2 Warfarin adherence (measured by the Med‐emonitor) 3 Thromboembolism 4 Bleeding events
[24]	An interactive educational website that included content on stroke prevention, details on the risk factors involved, the role of medication in reducing the risk and lifestyle modifications	Care as chosen by a neurologist. No access to the interactive/website education	Potential adaptations to lifestyle (specifically the effects of smoking, diet and exercise) were discussed with participants	1 Adherence to warfarin, statins, antiplatelets, antihypertensives and antidiabetics for preventing stroke (measure not reported explicitly)	2 New onset of ischaemic stroke, new onset of haemorrhagic stroke, acute coronary syndrome and all‐cause death
[25]	The Med‐emonitor stored and dispensed warfarin, with encouraging/educational automated messages displayed on its screen. The reminder group had additional reminders/alarms. The lottery group could win $10 (one in five) or $100 (one in 100) daily upon warfarin intake. The remaining group had a combination of reminders and a daily lottery	Participants were provided with the Med‐emonitor, which had the same functions as the ones provided for the intervention groups (but without the reminders/alarms/lottery incentives)	No	1 Percentage of time outside INR range	2 Percentage of days with incorrect warfarin adherence (measured by the Med‐emonitor) 3 Bleeding, stroke and thrombotic events
[26]	The intervention was the ‘Banhu’ mobile app following heart valve replacement. Patients received digital educational materials, reminders, and remote communication from clinicians in addition to usual care	Standard care, which included routine care during hospitalization and routine follow‐up care	No	1 Quality of life (measured using a standardized quality‐of‐life scale) 2 Warfarin adherence (measured using the Morisky medication Adherence Scale) 3 INR value compliance rates (percentage of INR results within therapeutic range)	4 Self‐care ability (measured using the self‐care ability scale)
[27]	Medication adherence was determined by artificial intelligence software, which also provided dosing instructions and reminders (sent within an hour of a missed dose or towards the end of the specified dosing time). Clinicians were alerted by intake errors in an automated manner via email/text	No details are reported explicitly	No	1 Adherence to warfarin, dabigatran, rivaroxaban or apixaban (measured by pill counts, visual confirmation of drug being taken via artificial intelligence and plasma sampling)	2 Activated partial thromboplastin time 3 Prothrombin time 4 Artificial intelligence usability and feasibility (measured by questionnaire)
[28]	The MyChart online portal allowed messages and reminders to be delivered to the participant upon an intake error. Educational material (such as risks and side effects) and video links could also be accessed in MyChart	Standard care. Medication was dispensed and stored using a smart pill bottle	No	Do not state which outcome was the primary outcome	Medication adherence for warfarin and DOACs (measured using a smart pill bottle) Intervention uptake (measured by MyChart logins) AF knowledge (measured by a series of questions)
[29]	An electronic OAC decision support tool was used to facilitate a cardiac nurse‐led session, encouraging suitable OAC use. Three to four telephone follow‐up calls were conducted by a cardiac nurse where progress was reviewed (three calls would be attempted). The length of the calls is Not reported explicitly	Usual cardiologist/general practitioner follow‐up care, who was responsible for determining the frequency of follow‐up	The cardiac nurse‐led session assessed risk factors and discussed medication management. Motivational interviewing for goal setting was employed with resources printed/provided in a written format for the participants. The session duration was about 1 h (but varied based on each participant)	1 HRQL (measured by the SF‐12 questionnaire)	2 Medication adherence for warfarin and DOACs (measured using the Morisky medication adherence scale) 3 Cardiovascular risk factors (BP, BMI, waist circumference, and physical activity) measured by conducting measurements and by using the global physical activity questionnaire 4 Appropriate use of OAC (measured by the CHA_2_DS_2_‐VASc where a score of ≥2 is indicative of OAC use unless a contradiction is present, 0 is indicative of OAC not being necessary expect in certain scenarios and 1 indicates that OAC use is appropriate unless being female was an isolated risk factor)
[30]	Participants received smartphones with both the Kardia application and an animated counsellor application installed. The Kardia application measured participant HR and rhythm, with readings transferred to a dashboard for the study team to view. The smartphone‐based animated counsellor application interacted with participants through conversations. The content of these conversations included education, symptoms and adherence. Daily use was instructed	The control group received a smartphone with a general health application aimed at improving health‐related knowledge. The app also included anticoagulation and atrial fibrillation material developed by the American Heart Association	No	1 Adherence to anticoagulation at 12 months (measured by pharmacy refills and a 3‐item validated instrument)	2 Quality of life (measured with the disease‐specific Atrial Fibrillation Effect on Quality of Life and the population‐standardized Patient‐Reported Outcomes Measurement Information System) 3 Healthcare utilization (measured by self‐report and review of the regional healthcare system's longitudinal electronic health record)
[31]	Smartphones were provided to study participants which had the pre‐installed Kardia application to measure their HR and rhythm. The smartphone also had a smartphone‐based animated counsellor application pre‐installed. The counsellor interacted with participants, with conversation content focused on education, symptoms and adherence. Participants were encouraged to use both applications every day	The control group received a smartphone with a general health application that had anticoagulation and atrial fibrillation material developed by the American Heart Association. They also received the Kardia application to measure HR and rhythm	No	1 12‐month adherence to oral anticoagulation (measured by pharmacy medication claims)	2 Quality of life (measured with the disease‐specific Atrial Fibrillation Effect on Quality of Life and the population‐standardized Patient‐Reported Outcomes Measurement Information System) 3 Healthcare utilization (measured using self‐report of events followed by review of the electronic health record)
[32]	Follow‐up telephone calls by a clinical pharmacist were made post‐discharge every week for the 6‐month duration of the study (no further details regarding the content/duration of the calls are explicitly reported)	The usual guidance on medication, routine monitoring and education was provided by a clinical pharmacist. Telephone follow‐up calls made at 3‐ and 6‐month post‐discharge by a clinical pharmacist	Usual care that mirrored that of the control group	Do not state which outcome was the primary outcome	TTR Warfarin and rivaroxaban medication adherence (measured using a standardized form) Adverse events Patient satisfaction (measured by questionnaire)
[33]	An online OAC shared decision‐making tool used during an in‐person encounter. Information is provided by the tool such as the risks involved, the cost of the medication and possible interactions. The tool can be viewed and accessed online without any restrictions (no further details are explicitly reported)	Usual care (no further details are explicitly reported)	The encounter was in‐person	Primary outcomes (participant‐perceived quality of the shared decision‐making tool, measured by a survey) have been previously published^47^	Primary (measured by pharmacy refill data) and secondary (expressed as PDC for DOACs and TTR for warfarin) adherence Clinical safety outcomes
[34]	Patient training on an individual basis (and refresher training at week eight of the study) to access the online PHR (MyChart) via a mobile/computer device. MyChart allows participants to access educational material, communicate with clinicians, review results of tests, arrange/review appointments and manage prescriptions	Access to the PHR (MyChart) without any training or personalization	No	Did not state which outcome was the primary outcome	Dabigatran adherence (measured using pharmacy refill data) Dabigatran knowledge (measured by questionnaire, with higher scores indicative of better knowledge) Patient engagement (measured by the PAM survey, range of 0–100 with higher scores indicative of better engagement)
[35]	A MEMS cap was used to monitor medication adherence. During the feedback phase, clinicians conducted telephone calls if an ‘unprotected day’ occurred, defined as ≥3 (apixaban) or ≥1 (rivaroxaban) missed doses or excess doses during the previous 24 h	A MEMS cap was used to monitor participant medication adherence. Participants were required to position the MEMS onto a wireless reader following each dose intake for data to be transferred	No	Do not state which outcome was the primary outcome	Apixaban and rivaroxaban adherence (measured by a MEMS, pill count and MMAS‐8) Cost‐effectiveness Patient experience (measured by questionnaire)
[36]	Each participant chose a reminder tool. Access was provided to a virtual clinic, including telephone follow‐up within a week of group allocation to check understanding. Between 10–20 days later, another call was made. Calls were then conducted monthly to offer advice and stress the value of adhering to medication	Information about apixaban was provided by the investigator, which included both an information sheet and a card	Information about apixaban was provided by the investigator, which included both an information sheet and a card. Intervention participants received an educational booklet	1 Apixaban dosing regimen implementation adherence (measured using the Helping Hand)	2 Apixaban persistence 3 Clinical events
[37]	The Care4Today smartphone‐based application tracked adherence and refill activity while sending reminders. Text messages were sent (‘semi‐automated’), and telephone calls were made if medication ingestion was as low as 0, 1 or 2 days a week. If calls went unanswered, information was transferred to the participant via voicemail and text	Usual AF care was provided by the participant's primary AF clinician (who was the prescriber and responsible for medication refills)	No	1 Rivaroxaban adherence (measured by pill counts/refill data)	2 Proportion of participants with PDC ≥ 80% 3 Change in the MMAS‐8 scores 4 Medication persistence (measured by the proportion of participants with active prescription at 6 months)
[38]	Telephone follow‐up at seven, 21 and 90 days by a pharmacist that integrated the NMS programme. Participants were given the opportunity to ask questions during these calls. Reminders were sent via a smartphone‐based application, but it is Not reported explicitly whether these reminders were automated or triggered by nonadherence	Usual pharmacy care, with follow‐up telephone calls made by a pharmacist at seven, 21 and 90 days. The group could use any smartphone‐based application/reminder system if they wished to do so	A pharmacist educated the participants on the medication during dispensing. A dabigatran information sheet with pictures was provided	Dabigatran adherence (measured by questionnaire)	No other outcomes were assessed
[39]	The AliveCor Kardia is a smartphone‐compatible ECG monitor to measure heart rhythm. For 5 days a week, participants took a reading every day. An online portal was used to assess participant monitoring adherence, with portal‐based reminders sent by a nurse if participant nonadherence was evident	Standard AF treatment and monthly nurse visits, where the smartphone ECG was used to measure heart rhythm	Participants were instructed on the application by a nurse. Participants received a prefilled pill organizer	1 Apixaban adherence (measured by pill count)	2 Composite of deaths, stroke, and hospitalization for congestive heart failure/AF
[40]	The intervention consisted of a medication intake reminder system that included both acoustic and visual reminder alarms	No reminder	All participants received a pillbox and a pharmacist counselling session	1 100%‐timing adherence (all adherence outcomes were measured using the Time4Med™ Smart card)	2 Percentage of patients with 100%‐taking adherence 3 Overall timing adherence (proportion of doses taken within the grace interval as an absolute value) 4 Overall taking adherence (proportion of doses taken as an absolute value) 5 Occurrence of predefined clinical events of interest (recurrent ischaemic stroke, transient ischaemic attacks [TIA], intracranial haemorrhage, major extracranial haemorrhage, myocardial infarction, venous thromboembolism, death) 6 Patient satisfaction with the reminder (measured using a questionnaire)
[41]	6‐month behaviour‐change programme based on the theory of planned behaviour and nudge strategies, delivered both in‐hospital and after discharge. After hospital discharge, support was provided mainly through the WeChat app and telephone, including monthly 20–30‐min follow‐ups, educational articles and feedback	Usual care, which included oral anticoagulant education during hospitalization	A nurse completed the in‐hospital portion of the intervention	Did not state which outcome was the primary outcome	Medication adherence (measured using the MMAS‐8) Theory of Planned Behaviour Questionnaire Outcomes Quality of life
[42]	A custom smartphone app that delivered timed push alerts reminding patients to take their edoxaban dose and to measure their blood pressure and heart rate via Bluetooth, allowing both patients and clinicians to view health data	The control group received standard care and the same blood pressure monitor but no access to the app	No	1 Adherence to edoxaban at 3 and 6 months (measured using pill counts) 2 Adequate medication adherence (defined as ≥95%)	3 Clinical composite endpoints (including stroke, systemic embolic events, major bleeding requiring hospitalization or transfusion and death during the 6‐month follow‐up period) 4 Frequency of app use (measured using the input of vital signs into the app)
[43]	The smartphone‐based Kardia application measures HR and rhythm. The smartphone‐based animated counsellor interacts with the participant, with conversations focused on education, symptoms, and adherence. Dialogue is personalized based on previous conversations, using the participant's real name and details. Participants used both applications every day	Sole access to the application	No	1 HRQL (measured by the AFEQT instrument, 0–100 range with higher scores indicative of better HRQL)	2 Self‐reported adherence to anticoagulation. OAC N/S. (measured by questionnaire with lower values indicative of better adherence) 3 Intervention acceptability (qualitative)
[44]	Telephone calls were made at week 1, months 2 and 6, to reiterate the in‐person education that was provided upon discharge	Standard AF care	A discussion (between 15 and 20 min) regarding OAC treatment with a study team member upon discharge and a leaflet summarizing all the information provided. Motivational interviewing was a key aspect, aiming to motivate participants to adhere to their medications	1 OAC (N/S) adherence (measured by claimed prescriptions).	2 OAC persistence 3 Treatment gaps (continuous OAC use = no gaps/gaps <7 days, transient treatment gaps = 7–89 days and major treatment gaps = 3 months/more) 4 Clinical events (cardiovascular and thrombotic death, major bleeding, and myocardial infarction)
[45]	The Alfalfa App that features patient education, remote consultation and medication intake reminders	Routine care	All study participants received routine care at baseline. This included receiving information from the cardiologist during the clinic visit, as well as an information booklet containing information on both AF and OAC	Patient's knowledge (measure suing the Jessa Atrial Fibrillation Knowledge Questionnaire) Medication adherence (measured using the MMAS‐8) Medication satisfaction (measured using the Treatment Satisfaction Questionnaire for Medication version II) Clinical outcomes (including bleeding and thrombotic events)	All outcomes were described as primary outcomes
[46]	Participants received online education using the AF‐EduApp, covering aspects of integrated AF care, knowledge on AF and oral anticoagulation, reinforcement of medication adherence, awareness about risk factors and reachability. Additional telemonitoring‐based feedback was provided to those with adherence <80%	Standard care with no access to the online or in‐person educational materials	Brochure on AF	1 Cumulative occurrence of cardiovascular events (including cardiovascular death, unplanned cardiovascular hospitalizations, unplanned cardiovascular outpatient visits and emergency department visits for cardiovascular reasons)	2 AF‐related knowledge (measured by the Jessa Atrial fibrillation Knowledge Questionnaire) 3 Awareness and self‐management of risk factors 4 OAC adherence (measured using a MEMS cap) 5 Contacts with care team

Abbreviation: AF, atrial fibrillation; AFEQT, Atrial Fibrillation Effect on Quality of Life instrument; MI, body max index; BP, blood pressure; CHA_2_DS_2_‐VASc, congestive heart failure, hypertension, age ≥75 years, diabetes, stroke/transient ischaemic attack, thromboembolism, vascular disease, age 65–74 years, sex category; DOAC, direct oral anticoagulant; HRQL, health‐related quality of life; INR, international normalized ratio; MEMS, Medication Events Monitoring Systems; MMAS‐8, 8‐part Morisky Medication Adherence Survey; OAC, oral anticoagulation; OAC, oral anticoagulation; PAM, Patient Activation Measure; PDC, proportion of days covered; PHR, personal health record; SF; Short Form 12 survey; TTR, time in therapeutic range.

### 3.3 mHealth interventions

Seven different mHealth interventions were described within the included studies, with eight studies including an element of personalization^27^, ^28^, ^29^, ^33^, ^34^, ^35^, ^39^, ^43^ and 14 using a multicomponent mHealth intervention.^25^, ^26^, ^27^, ^28^, ^29^, ^35^, ^36^, ^37^, ^38^, ^39^, ^41^, ^42^, ^45^, ^46^ The mHealth interventions most frequently employed were app‐based interventions.^26^, ^30^, ^31^, ^37^, ^38^, ^39^, ^41^, ^42^, ^43^, ^45^, ^46^ Patients were called by telephone to review their progress, provide information or answer queries. In four studies, telephone calls and text messages were conducted or sent in response to episodes of non‐adherence, acting as reminders for participants to take their medication.^27^, ^35^, ^37^, ^46^ Other methods of electronic reminders included alerts and notifications, including via smartphone‐based applications and online portals.^25^, ^26^, ^27^, ^28^, ^36^, ^37^, ^38^, ^39^, ^40^, ^42^, ^45^ Web‐based platforms were used to promote patient education and healthcare management in four studies.^24^, ^28^, ^34^, ^39^ RCTs that assessed smartphone‐based applications and electronic medication dispensers or caps included two that additionally incorporated lottery‐based incentives into the technical design of the electronic dispenser.^23^, ^25^ Other trials assessed shared decision‐making OAC support tools^29^, ^33^ and an artificial intelligence platform.^27^

Most studies reported usual or standard care as the comparator group.

### 3.4 Subgroup analyses

Six of the nine studies examining DOACs exclusively demonstrated statistically significant improvements in adherence,^34^, ^35^, ^38^, ^40^, ^41^, ^42^ contrasting with only one of the seven studies assessing both DOACs and warfarin reporting a significant effect^32^ and two of the four studies investigating warfarin alone.^25^, ^26^ Cross‐study comparisons based on disease or clinical indication for OAC, and dosing frequency were limited, as most studies were in patients with atrial fibrillation (18/24) and few provided explicit dosing information (6/24). Even when typical dosing frequencies were inferred from the specific anticoagulants used, no consistent effect on adherence were observed.

### 3.5 Definition and measurement of adherence

Just one study reported explicitly the adherence phase studied (implementation).^36^ A range of methods for measuring adherence were used across the studies—these are presented in Table 3. Electronic measures, such as smart pill bottles and medication events monitoring systems, were the most frequently employed. Other methods included self‐reported measures of adherence (questionnaires and standardized forms), pharmacy data (dispensing and refill data), artificial intelligence determination of dose‐taking, plasma sampling and pill counts. One study did not specify the adherence measure used,^24^ six studies did not report the metric used to quantify adherence,^24^, ^29^, ^32^, ^39^, ^40^, ^46^ and seven studies did not report the method of data aggregation used to summarize the adherence data collected.^23^, ^28^, ^34^, ^35^, ^38^, ^40^, ^46^

**TABLE 3:** Adherence characteristics.

References	Phase	Measurement	Metric	Data aggregation
[23]	Not reported explicitly	The Med‐emonitor (a MEMS)	Percentage of days incorrect	Not reported explicitly
[24]	Not reported explicitly	Not reported explicitly	Not reported explicitly	The number of adherent participants, as determined by the SMART programmes (details unspecified), expressed as a percentage of the total number of participants in the group, for each group
[25]	Not reported explicitly	The Med‐emonitor (a MEMS)	Percentage of days that adherence was incorrect	Median (IQR) for each group
[26]	Not reported explicitly	MMAS‐8	MMAS‐8 scoring system not reported explicitly	Mean (SD) for each group
[27]	Not reported explicitly	Pill counts, visual confirmation of drug being taken via artificial intelligence and plasma sampling	The pill count metric is not reported explicitly Plasma samples were described as ‘adherent’ if the minimum therapeutic range (which is not reported explicitly) was reached	Mean cumulative adherence per patient (pill count, artificial intelligence) The mean number of ‘adherent’ samples, expressed as a percentage of the total number of samples (plasma sampling)
[28]	Not reported explicitly	Smart pill bottle	The number of doses taken as a proportion of the number of prescribed doses	Not reported explicitly
[29]	Not reported explicitly	Morisky medication adherence scale	Not reported explicitly	The number of participants with ‘low’, ‘medium’ and ‘high’ scores, expressed as a percentage of the total number of participants in the group, for each group
[30]	Not reported explicitly	Pharmacy records of prescription fills and a 3‐item validated instrument	The proportion of days covered The validated instrument's scoring system is outlined (participants who scored <5 on the first item or >1 on the other items considered non‐adherent)	Median (IQR)
[31]	Not reported explicitly	Pharmacy medication claims	Proportion of days covered	Mean (SD)
[32]	Not reported explicitly	Standardized form	Not reported explicitly	The number of cases in the same period, self‐reduction of medication frequency and missed and low doses, expressed as a percentage of the total number of participants in the group, for each group
[33]	Not reported explicitly	Pharmacy refill data	The number of prescriptions filled (primary adherence), the proportion of days covered for DOACs and time spent in the therapeutic range for warfarin (secondary adherence)	The number of participants who filled their first prescription, expressed as a percentage of the total number of participants in the group, for each group (primary adherence) Mean for each group (secondary adherence)
[34]	Not reported explicitly	Pharmacy dispensing and refill data	Medication possession ratio	Not reported explicitly
[35]	Not reported explicitly	MEMS, pill count and MMAS‐8	The proportion of prescribed doses taken (taking adherence) and the proportion of days with the correct number of doses taken (regimen adherence) are established by MEMS data. MMAS‐8 scoring system nor pill count metric explicitly reported	Not reported explicitly
[36]	Implementation	Electronic monitoring device (the Helping Hand)	The proportion of days adhering to the prescribed regimen	Mean (SD) for each group
[37]	Not reported explicitly	Pill counts and pharmacy refill data	The proportion of days covered	Mean (SD) and median (IQR) for each group
[38]	Not reported explicitly	Questionnaire	The questions are outlined	Not reported explicitly
[39]	Not reported explicitly	Pill counts	Not reported explicitly	Median (IQR) for each group
[40]	Not reported explicitly	Time4Med™ smart card (electronic monitoring)	Not reported explicitly	Not reported explicitly
[41]	Not reported explicitly	MMAS‐8	MMAS‐8 scoring system outlined (a higher score indicates better adherence)	Not reported explicitly
[42]	Not reported explicitly	Pill counts	Medication possession ratio	Median (IQR)
[43]	Not reported explicitly	Questionnaire	The questions are outlined	The number of participants answering ‘yes’, expressed as a percentage of the total number of participants in each group, for each question
[44]	Not reported explicitly	Claimed prescriptions	The proportion of days covered (‘good adherence’ defined as PDC > 80%)	The number of ‘good adherence’ events, expressed as a percentage of the total number of participants in each group, for each group
[45]	Not reported explicitly	MMAS‐8	MMAS‐8 scoring system outlined	Median (IQR)
[46]	Not reported explicitly	Pill bottle smart caps (a MEMS)	Not reported explicitly	Not reported explicitly

Abbreviations: DOACs, direct oral anticoagulants; IQR, interquartile range; MEMS, Medical Events Monitoring System; MMAS‐8, 8‐part Morisky Medication Adherence Survey; PDC, proportion of days covered; SD, standard Deviation.

### 3.6 Summary of findings

Thirteen studies reported a significant improvement in medication adherence (Table 4). Telephone calls or text messages and smartphone‐based apps were most frequently associated with statistically significant improvements in medication adherence.^26^, ^32^, ^35^, ^38^, ^41^, ^42^, ^44^, ^45^, ^46^ For instance, one of the largest studies included in this review reported mean (SD) OAC adherence (based on percentage of days covered >80% derived from 1 year of prescriptions claims data), as 0.85 (±0.26) in the intervention group, compared with 0.75 (±0.31) in the control group (*p* < .001).^44^ Eight studies reporting a significant improvement used an intervention that was consisted of multiple components of mHealth.^25^, ^26^, ^35^, ^38^, ^41^, ^42^, ^45^, ^46^ In the study combining the most components of mHealth (app, reminders and telephone calls or texts), Merks et al^38^ described self‐reported adherence to the prescribed dose at 90 days as 78.4% in the intervention group, compared with 39.7% in the control group, a difference of 38.7 percentage points (OR 5.51, 95% CI 3.08–9.85, *p* = .0001). Electronic medication dispensers or smart pill bottles were used in four studies that indicated statistical significance.^25^, ^35^, ^40^, ^46^ Kimmel et al^25^ additionally incorporated lottery incentives into the technical design of the dispenser and reported an 11.6% (95% CI −14%, −0.3%) difference in the percentage of days with incorrect warfarin adherence between lottery (12.1%) and control (23.7%).

**TABLE 4:** Summary of the findings of each study.

References	Primary outcome results and significance (intervention *vs.* comparator)	Secondary outcome(s) results and significance (intervention *vs.* comparator)	Authors' conclusion
[23]	1 Overall warfarin anticoagulation control: 23.0% *vs*. 25.9% (unadjusted OR 0.81, 95% CI 0.53–1.22, *p* = .3051)	2 Overall warfarin adherence (percentages interpreted from fig. 2B of the study): 18% *vs*. 23% (unadjusted OR 0.78, 95% CI 0.49–1.25, *p* = .3038) 3 No thromboembolism 4 No bleeding that required hospitalization but 3 ED visits in the intervention group and 17 minor bleedings in the intervention group *vs*. 10 minor bleedings in the control group (*p* = .36)	Overall anticoagulation control was not significantly impacted by the Med‐emonitor's lottery intervention. A significant impact was only seen in a small subgroup of ‘high risk for poor adherence’ participants during post hoc analysis
[24]	1 Adherence at 12 months: Statins 56% *vs*. 33% (*p* = .006) Antiplatelet 81% *vs*. 75% (*p* = .088) Antihypertensive 67% *vs*. 69% (*p* = .661) Antidiabetic 73% *vs*. 67% (*p* = .297) No percentages or significance values were reported for warfarin due to the small number of participants	2 Composite secondary endpoints at 12 months: 3.50% *vs*. 3.59% (*p* = .921)	The website‐based educational intervention programme was proven to be feasible but failed to significantly improve adherence rates and outcomes
[25]	1 Percentage of time out of INR range: 30.1% (lottery) *vs*. 23.8% (reminder) *vs*. 23.9% (lottery + reminder) *vs*. 31.6% (control) (*p* = .29)	2 Percentage of days with incorrect warfarin adherence: 12.1% (lottery) *vs*. 21.8% (reminder) *vs*. 17.6% (lottery + reminder) *vs*. 23.7% (control) (*p* = .03). Significance was only observed between lottery and control groups (95% CI −14%, −0.3%, but no *p* value explicitly reported) 3 Bleeding events: 6.2% (lottery) *vs*. 6.3% (reminder) *vs*. 7.6% (lottery + reminder) *vs*. 6.2% (control) (*p* = 1.00) One stroke (*p* = .76) and two thrombotic events (non‐CNS) in the lottery group (*p* = .18)	Reminders, but not lottery incentives alone or in combination with reminders, proved beneficial in improving warfarin control. Despite this, improved warfarin adherence was only observed in the lottery incentives group
[26]	1 Quality of life at 6 months: Physical functioning: 62.56 ± 1.89 *vs*. 82.32 ± 1.13 (*p* < .001) Social functioning: 72.67 ± 2.57 *vs*. 82.32 ± 2.39 (*p* = .008) Role‐physical: 45.93 ± 4.4 *vs*. 73.17 ± 2.81 (*p* < .001) Role‐emotional: 88.37 ± 2.91 *vs*. 99.19 ± 0.81 (*p* < .001) Mental health: 67.53 ± 1.60 *vs*. 75.41 ± 1.11 (*p* < .001) Vitality: 57.79 ± 1.66 *vs*. 71.59 ± 1.69 (*p* < .001) Bodily pain: 57.63 ± 1.93 *vs*. 68.29 ± 1.74 (*p* < .001) General health: 69.07 ± 1.90 *vs*. 76.95 ± 1.80 (*p* = .003) 2 Warfarin adherence at 6 months: 4.14 ± 0.23 *vs*. 9.29 ± 0.23 (*p* < .001) 3 INR value compliance rates at 6 months: 18 (41.86) *vs*. 35 (85.37) (*p* < .001)	Self‐care ability at 6 months: 45.88 ± 0.76 *vs*. 57.07 ± 0.41 (*p* < .001)	The ‘banhu’ app significantly improved medication adherence, INR control, self‐care ability and quality of life in patients after mechanical heart valve replacement surgery
[27]	1 Pill count mean cumulative adherence: 97.2% (SD 4.4%) *vs*. 90.6% (SD 5.8%) Plasma sample adherence: 100% *vs*. 50% Artificial intelligence mean cumulative adherence: 90.5% (SD 7.5%) Significance level not reported	2 Activated partial thromboplastin time: 41.7 *vs*. 48.4 3 Prothrombin time: 35.1 *vs*. 32.9 4 INR: 3.4 *vs*. 3.1 5 Artificial intelligence usability and feasibility: 73.3% (pre‐study) and 83.3% (post‐study) rated the intervention ‘extremely good’ Significance level not reported	Medication adherence was increased by the intervention. This highlights the potential benefit of real‐time monitoring for promoting DOAC treatment behavioural changes
[28]	Primary outcome not stated	Overall warfarin and DOAC adherence: 93.1% *vs*. 89.5% (95% CI ‐0.04‐0.002, *p* = .08) Intervention uptake during the study: 42.7 ± 37.0 *vs*. 15.9 ± 16.0 (*χ* ^2^ = 62.04, *p* < .0001) AF knowledge at follow‐up was higher in the intervention than in the control group (*χ* ^2^ = 6.66, *p* = .0099, controlled for AF knowledge at baseline), but no percentages were explicitly reported	Both medication adherence and AF knowledge were increased by the intervention. This illustrates the feasibility of using health‐promoting technologies in mature adults
[29]	1 HRQL at 3 months: Physical component summary 51 *vs*. 45 (*p* = .03) Mental component summary 53 *vs*. 52 (*p* = .42) Physical functioning 75 *vs*. 50 (*p* = .04) Role physical 75 *vs*. 50 (*p* = .01) Bodily pain 75 *vs*. 50 (*p* = .12) General health 60 *vs*. 60 (*p* = .17) Vitality 50 *vs*. 38 (*p* = .19) Social functioning 75 *vs*. 75 (*p* = .44) Role emotional 88 *vs*. 75 (*p* = .16) Mental health 75 *vs*. 63 (*p* = .19)	2 Warfarin and DOAC adherence at 3 months: No significant differences between intervention and control groups (*p* = .81), but no overall percentages were explicitly reported 3 Cardiovascular risk factors at 3 months: Systolic BP 125 ± 21 *vs*. 124 ± 15 mmHg (*p* = .80) Diastolic BP 71 ± 11 *vs*. 73 ± 11 mmHg (*p* = .39) BMI 30.0 ± 6.7 *vs*. 30.2 ± 5.9 kg/m^2^ (*p* = .90) Waist circumference 104 ± 13 *vs*. 104 ± 16 cm (*p* = .97) Physical activity levels 8.5 ± 1.1 *vs*. 8.4 ± 1.2 (*p* = .60) Smoking status 6.1% *vs*. 3.0% (*p* = 1.0) 4 Appropriate OAC use at 3 months: 93.9% *vs*. 97.0% (*p* = 1.0)	No significant impacts were seen at follow‐up succeeding intervention implementation. This suggests that greater intervention intensity is needed when moving forward
[30]	1 Adherence to anticoagulation at 12 months: Pharmacy records of prescription refills: No significant difference between groups 0.97 (0.89–1.00) *vs*. 0.97 (0.92–1.00) or in PDC ≥ 0.80 Self‐reported adherence: Not significantly higher in the intervention group at 12 months	2 Patient‐Reported Outcomes Measurement Information System: No significant differences reported 3 Healthcare utilization: No significant differences reported	No statistically significant improvements in 12‐month anticoagulation adherence were observed
[31]	1 12‐month adherence to oral anticoagulation: 0.88 (95% CI 0.84–0.93) *vs*. 0.85 (95% CI 0.81–0.90). Not statistically significant	2 Quality of life: Disease‐specific quality of life at 12 months: Mean difference in scores between groups 0.34 (95% CI, −3.77–4.45). Not statistically significant General quality of life: No significant differences between groups at 4 and 8 months 3 Healthcare utilization: Emergency room visits and hospitalizations: 0.2 (95% CI 0.46–1.12) *vs*. 1.58 (95% CI 1.04–2.38). Statistically significant Total days hospitalized: 5.18 (95% CI 2.76–9.72) *vs*. 13.96 (95% CI 7.64–25.48). Statistically significant Serious adverse events: 44 *vs*. 59 (no statistical comparison reported)	The study failed to achieve its primary outcome of improved adherence to oral anticoagulation but achieved decreased healthcare utilization
[32]	Primary outcome not stated	1 TTR and proportion of patients with a TTR ≥ 65%: 73.22% ± 11.32, 77.5% *vs*. 60.15% ± 14.75, 44.9% (*p* < .05) 2 Medication adherence: Warfarin and rivaroxaban adherence was higher in the intervention group (*p* < .05) but no overall percentages were explicitly reported 3 Adverse events: Haemorrhagic stroke 1.64% *vs*. 12.5% (*p* = .019) Minor haemorrhage events 11.8% *vs*. 31.3% (*p* = .007). Thrombosis events 0 *vs*. 7.8% (*p* = .033) 4 Patient satisfaction: General satisfaction 4.1 ± 0.81 *vs*. 3.6 ± 0.91 (*p* = .041) Follow‐up technical quality 4.1 ± 0.68 *vs*. 3.6 ± 0.91 (*p* = .282) Follow‐up impersonal manner 4.1 ± 0.74 *vs*. 3.6 ± 0.69 (*p* = .019) Pharmacist patient communication 4.2 ± 0.72 *vs*. 3.5 ± 0.73 (*p* = .003) Follow‐up financial aspects 3.9 ± 0.73 *vs*. 3.6 ± 0.94 (*p* = .391) Follow‐up timeliness 4.1 ± 0.85 *vs*. 3.5 ± 1.02 (*p* = .048) Follow‐up accessibility and convenience 4.3 ± 0.67 *vs*. 3.7 ± 1.01 (*p* = .017)	The risk of haemorrhage and thrombosis became decreased, while the safety, effectiveness and adherence to warfarin and rivaroxaban became increased following a pharmacist‐led intervention
[33]	Primary outcomes published elsewhere	1 Primary adherence: 78% *vs*. 81% (95% CI 0.57–1.19) DOAC mean secondary adherence: 74.1 *vs*. 71.6 (95% CI −3.5–8.3) Warfarin mean secondary adherence: 66.6% (95% CI 61.9–71.4) *vs*. 64.4% (95% CI 42.8–54.1) 2 Combined major bleeding, cerebrovascular event, and any cause of death: 13% *vs*. 14%	Primary and secondary adherence were not significantly impacted, nor were the clinical safety outcomes following the implementation of a shared decision‐making tool
[34]	Primary outcome not stated	Dabigatran adherence: 97.47% ± 3.72 *vs*. 87.67% ± 14.48 (*p* = .012*) Knowledge score from baseline to 3 months: 3.75* ± 0.892 to 4.23 ± 0.912 (*p* = .005) *vs*. 3.70 ± 0.966 to 3.95 ± 0.846 (*p* = .124*) PAM score from baseline to 3 months: 63.03* ± 13.77 to 65.78* ± 13.92 (*p* = .078) *vs*. 63.08* ± 14.73 to 63.56* ± 11.25 (*p* = .814)	The study supports the use of the MyChart PHR for AF patient medication adherence, as well as for dabigatran knowledge improvement
[35]	Primary outcome not stated	1 Active phase adherence: Taking adherence 99.0% *vs*. 97.4% (*p* < .001) Regimen adherence 96.8% *vs*. 93.8% (*p* = .002) Pill count adherence 99.0% *vs*. 97.9% (*p* = .002) Observational phase adherence: Significant decreases were only observed in taking adherence (99.1%–94.3%, *p* = .049) and pill count adherence (99.1%–96.7%, *p* = .013). MMAS‐8 scores: 7.8 ± 0.4 (telemonitoring with feedback) *vs*. 7.4 ± 0.9 (telemonitoring) *vs*. 7.6 ± 0.5 (observational phase). No between‐group significance values were explicitly reported 2 Cost‐effectiveness: Incremental cost of the feedback to prevent one stroke €344 289, but estimates suggest that this could be reduced to €15, 488 by focusing on patients classed as high risk, that have low adherence, and by using technology that is optimized. 3 Patient‐reported experience: 87.2% said that the MEMS was practical 97.6% said that feedback was useful 63.8% said that they had an increased awareness of adherence	High rates of adherence were observed by implementing a telemonitoring intervention, with this effect being escalated upon adding the element of feedback. Focusing on high‐risk/low‐adherence cohorts could make the intervention more cost‐effective
[36]	1 Apixaban dosing regimen implementation adherence at 48 weeks: 90.4% ± 18.0 (continued intervention), 90.1% ± 18.6 (control), 89.3% ± 18.1 (secondary control) (*p* > .7)	2 Apixaban persistence at 48 weeks: 86.1% (continued intervention, 95% CI 81.3–89.7) *vs*. 85.2% (control, 95% CI 81.5–88.2) *vs*. 87.8% (secondary control, 95% CI 83.4–91.1) (*p* > .5) 3 Clinical events at 48 weeks: 4.8% (4.5% *vs*. 5.1%) of participants experienced at least one event which led to permanent discontinuation, but no *p* values were explicitly reported	Implementation of the intervention did not result in any additional significant benefit being observed, with adherence and persistence rates being high regardless of the intervention
[37]	1 Rivaroxaban adherence at 6 months: 0.86 ± 0.25 *vs*. 0.88 ± 0.25 (*p* = .62)	2 PDC ≥ 80% at 6 months: 91.9% *vs*. 85.1% (*p* = .62) 3 Change in MMAS‐8 from baseline to 6 months: 0.60 *vs*. 0.70 (*p* = .76) 4 Medication persistence: 70.8% *vs*. 82.1% (*p* = .12)	The intervention proved feasible, with no major differences observed between trial arms (with high adherence in both)
[38]	Adherence to prescribed dose at 90 days: 78.4% *vs*. 39.7% at (OR 5.51, 95% CI 3.08–9. 85, *p* = .0001) Adherence to daily usage at 90 days: 5.5 ± 1.3 *vs*. 4.4 ± 2.0 (*p* < .0001) Fully adherent patients at 90 days: 26.1% *vs*. 13.2% (OR = 2.32, 95% CI 1.18–4.56, *p* = .0145)	No other outcomes were assessed	Polish community pharmacy interventions (using smartphones and pictograms) can lead to improved medication adherence
[39]	1 Apixaban adherence at 6 months: 100% *vs*. 99.7% (*p* = .247)	2 Composite secondary endpoints: 8 (SD 17.8) *vs*.10 (SD 20.4) (*p* = .067)	The intervention did not result in a significant improvement in medication adherence between groups (all participants had a higher than anticipated adherence rate)
[40]	1 100%‐timing adherence: 10 patients (12%) *vs*. 0 patients (*p* = .002)	2 100%‐taking adherence: 10 patients (12%) *vs*. 2 patients (*p* = .039) 3 Overall timing adherence: 96% *vs*. 92% 4 Overall taking adherence: 97% *vs*. 94% 5 Occurrence of predefined clinical events of interest: No statistically significant differences in clinical events between groups. Event numbers were low and the trial was not powered for clinical outcomes 6 Patient satisfaction with the reminder: Approximately half reported the reminder was impractical and not beneficial and 81% reported no restriction integrating the reminder into their daily routine	Intake reminder significantly increased timing adherence to DOAC and reduced the number of missed intakes in stroke patients
[41]	Primary outcome not stated	Medication adherence at 6 months: Significant differences in adherence between groups (*p* < .05) Theory of Planned Behaviour Questionnaire Outcomes at 6 months: attitude, subjective norm, intention, perceived behaviour control significant between groups (*p* < .05) Quality of life at 6 months: No significant differences between groups (*p* > .05)	A theory of planned behaviour programme and nudge strategy can significantly increase medication adherence in non‐valvular atrial fibrillation patients
[42]	1 Adherence to edoxaban: At 3 months: 8% (IQR 95–100) *vs*. 98% (IQR 91–100) (*p* = .06) At 6 months: 98% (IQR 94.5–100) *vs*. 97.5% (IQR 92.8–100) (*p* = .15) 2 Adequate medication adherence: At 3 months: 76.8% (146/190) *vs*. 64.7% (139/215) (*p* = .01). At 6 months: 73.9% (147/199) *vs*. 61.0% (136/223) (*p* = .007)	3 Clinical composite endpoints: No significant difference between groups 4 Frequency of app use: Median app use over 6 months: 65.6% (IQR 16.7–91.7) Participants using the app ≥50%: 57.5% (107/186) Higher app use showed a non‐significant trend towards better adherence	No overall difference in edoxaban adherence between groups was identified, although a higher proportion of patients in the intervention group achieved adequate adherence (defined as ≥95%)
[43]	1 HRQL score from baseline to 30 days: 81.5 (SD 14.2) to 85.2 (SD 14.1) *vs*. 76.0 (SD 17.6) to 76.1 (SD 16.7) (adjusted mean difference 4.5, 95% CI 0.6–8.3, *p = *.03)	2 Adherence from baseline to 30 days: 27.9%–3.5% *vs*. 22%–23.2% (adjusted difference 16.6%; 95% CI 2.8%–30.4%, *p* < .001)	Both medication adherence and HRQL were significantly improved using the intervention, with patients also accepting the intervention to a favourable level
[44]	1 OAC (N/S) adherence at 1 year: 0.85 ± 0.26 *vs*. 0.75 ± 0.31 (*p* < .001)	2 OAC persistence at 1 year: 88.2% *vs*. 75.6% (adjusted odds ratio of 2.42, 95% CI 1.72–3.41, *p* < .001) 3 Treatment gaps: Continuous OAC use 59.6% *vs*. 41.8% (95% CI 1.60–2.67, *p* < .001) Transient treatment gap 23.6% *vs*. 26.9% (95% CI 0.88–1.57, *p* = .275) Major treatment gap 16.8% *vs*. 31.2% (95% CI 1.76–3.26, *p* < .001) 4. Clinical events: All‐cause death 27.4% *vs*. 29.5% (95% CI 0.75–1.19, *p* = .605) Cardiovascular death 19.4% *vs*. 22.2% (95% CI 0.67–1.16, *p* = .361) Thromboembolic death 3.9% *vs*. 4.8% (95% CI 0.43–1.63, *p* = .600) Major bleeding 4.1% *vs*. 5.0% (95% CI 0.43–1.56, *p* = .545) Myocardial infarction 1.4% *vs*. 2.0% (95% CI 0.28–2.50, *p* = .259)	Motivational interviewing during OAC treatment for non‐valvular AF can improve adherence, reduce treatment gaps and increase persistence to a significant level
[45]	1 Patient's knowledge at 3 months: 78.1% (68.8%–92.2%) *vs*. 25.0% (12.5%–48.4%) (*p* < .001) 2 Medication adherence at 3 months: 8.0 (7.0–8.0) *vs*. 5.5 (4.5–6.8) (*p* < .001) 3 Medication satisfaction: Effectiveness 66.7 (50.0, 83.3) *vs*. 50.0 (50.0, 58.3) (*p* < .001) Side effects 100.0 (91.7, 100.0) *vs*. 91.7 (83.3, 100) (*p* = .006) Convenience 66.7 (55.6, 81.9) *vs*. 55.6 (50.0, 66.7) (*p* < .001) Global satisfaction 66.7 (58.3,83.3) *vs*. 54.2 (50.0, 66.7) (*p* < .001) 4 Clinical outcomes: No significant differences were observed between groups for bleeding or thrombosis over 12 months	All outcomes were described as primary outcomes	Anticoagulation knowledge, adherence and patient satisfaction were significantly increased after implementation of the Alfalfa App in AF patients
[46]	1 Composite cardiovascular events: The number of patients experiencing ≥1 cardiovascular event: 43.2% (in‐person) *vs*. 49.0% (online) *vs*. 45.7% (standard care) (*p* = .307) There were no statistically significant differences between groups for any of the cardiovascular endpoints	2 Knowledge scores at 18 months: 86.5 ± 13.2% (in‐person) *vs*. 82.5 ± 19.3% (online) *vs*. 65.3 ± 16.6% (standard care). Significant differences for both intervention groups *vs*. standard care (*p* < .001) but no difference between in‐person and online (*p* = .175) 3 Awareness and self‐management of risk factors: Significant improvements in multiple risk‐factor awareness and management domains occurred in the education groups, while almost no improvement was observed in standard care. Physical inactivity remained the most difficult behaviour to modify across all groups 4 OAC adherence at 1 year: 95.4 ± 7.3% (in‐person) *vs*. 94.0 ± 10.8% (online) *vs*. 91.6 ± 14.0% (standard care). Significantly higher in the in‐person (*p* = .006) or online (*p* = .042) education groups than in the standard care group 5 Contacts with care team: 25.5% (in‐person) and 17.9% (online) *vs*. 2.9% (standard care) (*p* < .001)	In‐person and online education led to substantial improvements in atrial fibrillation knowledge and risk‐factor awareness. Medication adherence remained high in all groups, and the intervention did not reduce the incidence of the primary composite cardiovascular endpoint

Abbreviations: AF, atrial fibrillation; AFEQT, Atrial Fibrillation Effect on Quality of Life; BMI, body max index; BP, blood pressure; CHA_2_DS_2_‐VASc, congestive heart failure, hypertension, age ≥75 years, diabetes, stroke/transient ischaemic attack, thromboembolism, vascular disease, age 65–74 years, sex category; DOAC, direct oral anticoagulant; HRQL, health‐related quality of life; INR, international normalized ratio; MMAS‐8, 8‐part Morisky Medication Adherence Survey; OAC, oral anticoagulation; PAM, Patient Activation Measure; PDC, proportion of days covered; PHR, personal health record; TTR, time in therapeutic range.

Of the 24 included studies, 15 measured clinical outcomes,^23^, ^24^, ^25^, ^29^, ^30^, ^31^, ^32^, ^33^, ^36^, ^39^, ^40^, ^42^, ^44^, ^45^, ^46^ six reported on coagulation tests results,^23^, ^25^, ^26^, ^27^, ^32^, ^33^ and six measured health‐related quality of life.^26^, ^29^, ^30^, ^31^, ^41^, ^43^ Statistical significance for clinical or biochemical outcomes was reported by Liu et al^32^ for time in therapeutic range (TTR) (73.2% ± 11.3 *vs*. 60.2% ± 14.8, *p* = .014), proportion of patients with TTR ≥ 65% (77.5% *vs*. 44.9%, *p* = .002), haemorrhagic stroke (1.6% *vs*. 12.5%, *p* = .019), minor haemorrhage events (11.8% *vs*. 31.3%, *p* = .007) and thrombosis events (0.0% *vs*. 7.8%, *p* = .033). Statistical significance for the difference in health‐related quality of life score from baseline to 30 days was reported by Guhl et al^43^ (adjusted mean difference in the Atrial Fibrillation Effect on Quality of Life instrument of 4.5, 95% CI 0.6, 8.3, *p* = .03) and by Gallagher et al^29^ for the physical component summary (51 *vs*. 45, *p* = .03), physical functioning (75 *vs*. 50, *p* = .04) and role physical (75 *vs*. 50, *p* = .01) components of the Short Form 12 survey.

### 3.7 Methodological quality (risk of bias assessment)

Overall, the studies included in this review were of poor methodological quality (Table 5), with all studies deemed to be at a critical overall risk of bias. Generally, studies scored best with respect to randomization and blinding procedures and data analysis and interpretation. Studies scored worst in relation to study design and implementation of procedures and adherence outcome measurement and reporting.

**TABLE 5:** Quality of reporting assessment.

References	Domain 1: Bias related to study design and implementation of study procedures	Domain 2: Bias related to randomization and blinding procedures	Domain 3: Bias related to adherence outcome measurement and reporting	Domain 4: Bias related to data analysis and interpretation	Overall judgement
[23]	Critical	Low	Critical	High	Critical
[24]	Critical	High	Critical	Critical	Critical
[25]	Critical	Low	Critical	High	Critical
[26]	Critical	High	Critical	High	Critical
[27]	Critical	High	Critical	High	Critical
[28]	Critical	Moderate	Critical	High	Critical
[29]	Critical	High	Critical	High	Critical
[30]	Critical	Critical	Critical	High	Critical
[31]	Critical	Critical	Critical	High	Critical
[32]	Critical	Low	Critical	High	Critical
[33]	Critical	Critical	Critical	Critical	Critical
[34]	Critical	Critical	Critical	High	Critical
[35]	Critical	Critical	Critical	High	Critical
[36]	Critical	Low	Critical	High	Critical
[37]	Critical	Critical	Critical	Critical	Critical
[38]	Critical	Low	Critical	High	Critical
[39]	Critical	High	Critical	High	Critical
[40]	Critical	High	Critical	High	Critical
[41]	Critical	High	Critical	High	Critical
[42]	Critical	Critical	Critical	High	Critical
[43]	Critical	Critical	Critical	High	Critical
[44]	Critical	Low	Critical	High	Critical
[45]	Critical	High	Critical	High	Critical
[46]	Critical	Critical	Critical	High	Critical

## 4 DISCUSSION

This review identified 24 studies that randomized 11 150 participants to mHealth interventions (or control) to improve adherence to OACs. The results of these trials indicate the potential for mHealth interventions to be effective in promoting adherence; however, the quality of the trials was poor, both from a methodological perspective, increasing their risk of bias, and in terms of reporting.

The findings of our review align with a previous systematic review of smartphone‐based applications in the context of cardiovascular disease management,^48^ which also reported that the quality of most trials was poor, with just 2/16 trials being reported as being of fair quality.^49^, ^50^ Elsewhere, 8/24 trials included in a non‐overlapping review of digital health solutions for cardiovascular disease prevention,^13^ and 5/26 RCTs of mHealth interventions in medication adherence^14^ were assessed as being of low risk of bias according to other tools.

Among the mHealth interventions included in our review, telephone calls or text messages and smartphone‐based apps were most frequently associated with statistically significantly improved medication adherence. This is consistent with the review by Bond et al^51^ in the context of adherence to statins, who concluded that to achieve the best results, multiple different mHealth interventions should be employed together. Despite this, our review identified that only eight of the 14 that used multiple components led to significant improvement in adherence.^25^, ^26^, ^35^, ^38^, ^41^, ^42^, ^45^, ^46^

The included studies varied substantially in their measurement of medication adherence, the types of mHealth interventions employed and the definitions of adherence outcomes. This heterogeneity made synthesis challenging, limiting our ability to directly compare results across studies and precluding meta‐analysis. Consequently, the overall strength and reliability of conclusions regarding the effectiveness of mHealth interventions are constrained. The evidence reviewed was insufficient to make an inference of intervention effects in specific subgroups. The trend towards effectiveness among studies of interventions to improve adherence to DOACs was negated by findings from studies that considered both DOACs and warfarin. The relationship between dose frequency and intervention effectiveness was confounded by incomplete reporting; and there was no suggestion that disease or clinical indication influenced the statistical significance of trial findings.

The detailed search strategy as well as the rigorous screening and selection process performed supports our contention that the reviewing methods used were robust and likely to not have missed many relevant studies. Distinct strengths include our application of the adherence‐specific RoBIAS tool to assess bias^16^ and rigorous critique of the methods of adherence measurement, analysis and reporting. It should be acknowledged, however, that limiting the inclusion criteria to only studies published in the English language may have excluded some trials. Additionally, study selection and data extraction were performed by a single reviewer, which may introduce bias.

Generally, the quality of the studies identified was poor, and the results were liable to bias. The lack of methodological rigour was apparent, as was the incompleteness of reporting. The description of the measure, metric and method of data aggregation of adherence data, for instance, was complete for only one study.^35^ Study sample sizes were often small and not calculated formally, suggesting that many trials were underpowered—certainly for the evaluation of clinical outcomes. Follow‐up periods were relatively short, with no trial following up patients for more than 1 year, which limits evaluation of the impact of mHealth interventions on persistence and longer‐term outcomes. The need for more robust research methods to improve the design, conduct and reporting of clinical trials that investigate medication adherence has been highlighted previously,^52^, ^53^ and improvements in these would allow for more definitive conclusion that the observed effects are solely due to the mHealth intervention that was tested.

Participants of the included studies were relatively comparable in age and had OAC treatment prescribed for similar clinical indications. Differences in the adherence rates for once‐ and twice‐daily OACs,^54^ however, may have important implications concerning the effectiveness of the mHealth interventions. While all participants likely had their medications subsidized for the duration of the study, the behaviours and beliefs in relation to disease and treatment may differ for other contextual reasons, including socio‐economic and healthcare system factors.^55^

Most of the studies included in this review were conducted in high‐income countries with public, insurance‐funded or private healthcare systems. Consequently, the findings may have limited generalizability to low‐ and middle‐income countries or populations with restricted access to mHealth technologies. Differences in healthcare infrastructure, medication funding, cultural beliefs and digital literacy could influence the feasibility and effectiveness of mHealth interventions in these settings. Future research should examine how these interventions perform in diverse contexts to inform implementation in broader populations.

The importance of maintaining adherence is supported by studies that have sought to understand the clinical significance of non‐adherence to OACs, which report a higher likelihood of adverse health outcomes including stroke and death.^56^ The hazard ratio for the risk of ischaemic stroke may be as high as double for non‐adherent (<80% doses taken) compared with adherent (≥ 80%) patients.^57^, ^58^, ^59^ Each 10% reduction in the proportion of days covered is associated with a 5%–14% higher risk of stroke, systemic embolism, transient ischaemic attack or death.^59^, ^60^, ^61^ Risk is dependent on patients' baseline CHA_2_DS_2_‐VASc score and increases with extended periods of dose interruption.^62^

## 5 CONCLUSIONS

This review suggests that mHealth interventions may be effective in improving OAC adherence in adults—with more evidence favouring smartphone‐based apps, telephone calls and messages for follow‐up support or as medication dosing reminders. However, all included studies were at high or critical risk of bias, with small sample sizes, short follow‐up periods and heterogeneous intervention types and adherence measures, somewhat limiting confidence in these findings. The apparent benefits of mHealth interventions should therefore be interpreted cautiously. Future research should focus on optimizing communication and messaging within the specific context studied—defining the optimal frequency of delivery, content and scope for automation as well as the use of artificial intelligence. Given the rapid advances in mHealth technologies, there will likely be more sophisticated mHealth solutions that may be linked to different forms of delivery and/or frequency. Personal telephone calls are likely to decline as a scalable and/or preferred option. Research should also focus on conducting larger, longer‐term trials that are of better methodological quality. Improved methodological rigour needs to be applied in the design, conduct and reporting of clinical trials involving mHealth interventions to improve medication adherence.

## AUTHOR CONTRIBUTIONS

Dyfrig A. Hughes conceived the research; Non Davies conducted the literature search and wrote the first draft of the manuscript; all authors made substantial contributions to the design and interpretation of the research, revised the manuscript for important intellectual content and approved the final version. All authors agree to be accountable for all aspects of the work in ensuring that questions related to the accuracy or integrity of any part of the work are appropriately investigated and resolved.

## CONFLICT OF INTEREST STATEMENT

Elin Haf Davies is Founder and CEO of Aparito, a wholly owned subsidiary of Eli Lilly. Dyfrig A. Hughes and Non Davies declare no conflicts of interest.

## Supporting information


**Appendix S1.** Supporting Information.

## Data Availability

Data are publicly available via electronic database searches.

## References

[1] Ageno W , Caramelli B , Donadini MP , Girardi L , Riva N . Changes in the landscape of anticoagulation: a focus on direct oral anticoagulants. Lancet Haematol. 2024;11(12):e938‐e950. doi:10.1016/S2352-3026(24)00281-3 39433055

[2] Ozaki AF , Choi AS , Le QT , et al. Real‐world adherence and persistence to direct oral anticoagulants in patients with atrial fibrillation: a systematic review and meta‐analysis. Circ Cardiovasc Qual Outcomes. 2020;13(3):e005969. doi:10.1161/CIRCOUTCOMES.119.005969 32148102

[3] Quirós López R , Formiga Pérez F , Beyer‐Westendorf J . Adherence and persistence with direct oral anticoagulants by dose regimen: a systematic review. Brit J Clinical Pharma. 2025;91(14):1096‐1113. doi:10.1002/bcp.70003 PMC11992665 39957057

[4] Tarn DM , Shih KJ , Schwartz JB . Reasons for nonadherence to the direct oral anticoagulant apixaban for atrial fibrillation. J Am Geriatr Soc. 2021;69(12):3683‐8687. doi:10.1111/jgs.17423 PMC8649070 34453325

[5] Vrijens B , De Geest S , Hughes DA , et al. A new taxonomy for describing and defining adherence to medications. Br J Clin Pharmacol. 2012;73(5):691‐705. doi:10.1111/j.1365-2125.2012.04167.x PMC3403197 22486599

[6] The World Health Organization . Adherence to long‐term therapies: evidence for action [Internet]. World Health Organization. 2003. Available from: https://apps.who.int/iris/handle/10665/42682

[7] Kleinsinger F . The unmet challenge of medication nonadherence. Perm J. 2018;22:18‐033. doi:10.7812/TPP/18-033 PMC6045499 30005722

[8] Bartoli‐Abdou JK , Patel JP , Crawshaw J , et al. Exploration of adherence and patient experiences with DOACs one year after switching from vitamin‐K antagonists – insights from the switching study. Thromb Res. 2018;162:62‐68. doi:10.1016/j.thromres.2017.12.021 29306122

[9] Gandapur Y , Kianoush S , Kelli HM , et al. The role of mHealth for improving medication adherence in patients with cardiovascular disease: a systematic review. Eur Heart J Qual Care Clin Outcomes. 2016;2(4):237‐244. doi:10.1093/ehjqcco/qcw018 PMC5862021 29474713

[10] Bradway M , Carrion C , Vallespin B , et al. mHealth assessment: conceptualization of a global framework. JMIR Mhealth Uhealth. 2017;5(5):e60. doi:10.2196/mhealth.7291 PMC5434253 28465282

[11] Dauletbaev N , Kuhn S , Holtz S , et al. Implementation and use of mHealth home telemonitoring in adults with acute COVID‐19 infection: a scoping review protocol. BMJ Open. 2021;11(9):e053819. doi:10.1136/bmjopen-2021-053819 PMC8478582 34580103

[12] Xu H , Long H . The effect of smartphone app‐based interventions for patients with hypertension: systematic review and meta‐analysis. JMIR Mhealth Uhealth. 2020;8(10):e21759. doi:10.2196/21759 PMC7605981 33074161

[13] Qi Y , Mohamad E , Azlan AA , Zhang C , Ma Y , Wu A . Digital health solutions for cardiovascular disease prevention: systematic review. J Med Internet Res. 2025;27:e64981. doi:10.2196/64981 PMC11803337 39847411

[14] Kim SK , Park SY , Hwang HR , Moon SH , Park JW . Effectiveness of mobile health intervention in medication adherence: a systematic review and meta‐analysis. J Med Syst. 2025;49(1):13. doi:10.1007/s10916-024-02135-2 39821698

[15] Sinnappah KA , Hughes DA , Stocker SL , Vrijens B , Aronson JK , Wright DFB . A framework for understanding sources of bias in medication adherence research. Br J Clin Pharmacol. 2023;89(11):3444‐3453. doi:10.1111/bcp.15863 37496213

[16] Sinnappah KA , Hughes DA , Stocker SL , Wright DFB , ESPACOMP the International Society for Medication Adherence . Risk of bias tools for medication adherence research: RoBIAS and RoBOAS. Br J Clin Pharmacol. 2025;91(5):1457‐1478. 10.1111/bcp.16382 PMC12035589 39837562

[17] De Geest S , Zullig LL , Dunbar‐Jacob J , et al. ESPACOMP medication adherence reporting guideline (EMERGE). Ann Intern Med. 2018;169(1):30‐35. doi:10.7326/M18-0543 PMC7643841 29946690

[18] Dima AL , Allemann SS , Dunbar‐Jacob J , Hughes DA , Vrijens B , Wilson IB . TEOS: a framework for constructing operational definitions of medication adherence based on timelines–events–objectives–sources. Brit J Clinical Pharma. 2021;87(6):2521‐2533. doi:10.1111/bcp.14659 33220097

[19] Dima AL , Allemann SS , Dunbar‐Jacob J , Hughes DA , Vrijens B , Wilson IB . Methodological considerations on estimating medication adherence from self‐report, electronic monitoring and electronic healthcare databases using the TEOS framework. Br J Clin Pharmacol. 2023;89(7):1918‐1927. doi:10.1111/bcp.15375 35491721

[20] Campbell M , McKenzie JE , Sowden A , et al. Synthesis without meta‐analysis (SWiM) in systematic reviews: reporting guideline. BMJ. 2020;368:l6890. doi:10.1136/bmj.l6890 PMC7190266 31948937

[21] Page MJ , McKenzie JE , Bossuyt PM , et al. The PRISMA 2020 statement: an updated guideline for reporting systematic reviews. BMJ. 2021;372:n71. doi:10.1136/bmj.n71 PMC8005924 33782057

[22] Alexander SPH , Gibb AJ , Kelly E , et al. The concise guide to PHARMACOLOGY 2025/26: introduction and other protein targets. Br J Pharmacol. 2025;182(Suppl 1):S1‐S23. doi:10.1111/bph.70229 PMC13480412 41461576

[23] Kimmel SE , Troxel AB , Loewenstein G , et al. Randomized trial of lottery‐based incentives to improve warfarin adherence. Am Heart J. 2012;164(2):268‐274. doi:10.1016/j.ahj.2012.05.005 PMC4738867 22877814

[24] Peng B , Ni J , Anderson CS , et al. Implementation of a structured guideline‐based program for the secondary prevention of ischemic stroke in China. Stroke. 2014;45(2):515‐519. doi:10.1161/STROKEAHA.113.001424 24385269

[25] Kimmel SE , Troxel AB , French B , et al. A randomized trial of lottery‐based incentives and reminders to improve warfarin adherence: the warfarin incentives (WIN2) trial. Pharmacoepidemiol Drug Saf. 2016;25(11):1219‐1227. doi:10.1002/pds.4094 PMC5093038 27592594

[26] Ding N , Luo X , Zhou J , Jiang X , Wang X . Intervention effect of the mobile phone APP based continuous care on patients after mechanical heart valve replacement: a randomised controlled trials. Rev Cardiovasc Med. 2024;25(9):314. doi:10.31083/j.rcm2509314 PMC11440390 39355573

[27] Labovitz DL , Shafner L , Reyes Gil M , Virmani D , Hanina A . Using artificial intelligence to reduce the risk of nonadherence in patients on anticoagulation therapy. Stroke. 2017;48(5):1416‐1419. doi:10.1161/STROKEAHA.116.016281 PMC5432369 28386037

[28] Toscos T , Coupe A , Wagner S , et al. Engaging patients in atrial fibrillation management via digital health technology: the impact of tailored messaging. J Innov Card Rhythm Manag. 2020;11(8):4209‐4217. doi:10.19102/icrm.2020.110802 PMC7452738 32874747

[29] Gallagher C , Orchard J , Nyfort‐Hansen K , Sanders P , Neubeck L , Hendriks JM . Nurse led atrial fibrillation management: the NEAT study: a randomized controlled trial. J Cardiovasc Nurs. 2020;35(5):456‐467. doi:10.1097/JCN.0000000000000680 32251039

[30] Magnani JW , Plevniak K , Ferry D , et al. The mobile health intervention for rural patients with atrial fibrillation a randomized controlled trial. Int J Cardiol. 2025;438:133575. doi:10.1016/j.ijcard.2025.133575 PMC12974337 40609876

[31] Magnani J , Lalama C , Abebe K , et al. A mobile relational agent to enhance atrial fibrillation self‐care: primary and secondary outcomes of a randomized controlled trial. Am Heart J. 2025;290:115‐128. doi:10.1016/j.ahj.2025.06.009 PMC13382527 40545207

[32] Liu X , Xiao Q , Li Y , Hu G , Kun W , Xu W . Effect of post‐discharge pharmacist‐led follow‐up on drug treatment in patients with deep venous thrombosis in primary hospitals in China. Pak J Pharm Sci. 2022;35(3):785‐791. 35791477

[33] Noseworthy PA , Branda ME , Kunneman M , et al. Effect of shared decision‐making for stroke prevention on treatment adherence and safety outcomes in patients with atrial fibrillation: a randomized clinical trial. J Am Heart Assoc. 2022;11(2):e023048. doi:10.1161/JAHA.121.023048 PMC9238511 35023356

[34] Chen YC , Roebuck AE , Sami A , Ersin ÖH , Mirro MJ . The use of electronic personal health records to improve medication adherence and patient engagement: a randomized study of non‐valvular atrial fibrillation patients. J Innov Card Rhythm Manag. 2017;8(8):2804‐2813. doi:10.19102/icrm.2017.080803 PMC7252939 32494465

[35] Desteghe L , Vijgen J , Koopman P , et al. Telemonitoring‐based feedback improves adherence to non‐vitamin K antagonist oral anticoagulants intake in patients with atrial fibrillation. Eur Heart J. 2018;39(16):1394‐1403. doi:10.1093/eurheartj/ehx762 29300888

[36] Montalescot G , Brotons C , Cosyns B , et al. Educational impact on apixaban adherence in atrial fibrillation (the AEGEAN STUDY): a randomized clinical trial. Am J Cardiovasc Drugs. 2020;20(1):61‐71. doi:10.1007/s40256-019-00356-2 PMC6978445 31243691

[37] Turakhia M , Sundaram V , Smith SN , et al. Efficacy of a centralized, blended electronic, and human intervention to improve direct oral anticoagulant adherence: smartphones to improve rivaroxaban ADHEREnce in atrial fibrillation (SmartADHERE) a randomized clinical trial. Am Heart J. 2021;237:68‐78. doi:10.1016/j.ahj.2021.02.023 33676886

[38] Merks P , Cameron JD , Balcerzak M , et al. Evaluation of a pharmacist‐led intervention to improve medication adherence in patients initiating dabigatran treatment: a comparison with standard pharmacy practice in Poland. BMC Prim Care. 2022;23(1):210. doi:10.1186/s12875-022-01821-9 PMC9389729 35986241

[39] Tran AT , Okasha OM , Steinhaus DA , et al. Prospective evaluation of the effect of smartphone electrocardiogram usage on anticoagulant medication compliance. J Interv Card Electrophysiol. 2022;65(2):453‐460. doi:10.1007/s10840-022-01235-8 35513562

[40] Dietrich F , Polymeris A , Albert V , et al. Intake reminders are effective in enhancing adherence to direct oral anticoagulants in stroke patients – primary analysis of the MAAESTRO study. Eur Stroke J. 2023;8(2):184. doi:10.1177/23969873231169660 PMC10827905 37831125

[41] Ding Y , Jiang H , Liu J , Chen D , Yang F . Effects of the theory of planned behavior and nudge strategy‐based intervention on the adherence to anticoagulation treatment in patients with non‐valvular atrial fibrillation. Geriatr Nurs. 2023;51:17‐24. doi:10.1016/j.gerinurse.2023.01.023 36871327

[42] Yoon M , Lee JH , Kim IC , et al. Smartphone app for improving self‐awareness of adherence to edoxaban treatment in patients with atrial fibrillation (ADHERE‐app trial): randomized controlled trial. J Med Internet Res. 2024;26:e65010. doi:10.2196/65010 PMC11621717 39570659

[43] Guhl E , Althouse AD , Pusateri AM , et al. The atrial fibrillation health literacy information technology trial: pilot trial of a mobile health app for atrial fibrillation. JMIR Cardio. 2020;4(1):e17162. doi:10.2196/17162 PMC7501575 32886070

[44] Tzikas A , Samaras A , Kartas A , et al. Motivational interviewing to support oral anticoagulation adherence in patients with non‐valvular atrial fibrillation (MISOAC‐AF): a randomized clinical trial. Eur Heart J Cardiovasc Pharmacother. 2021;7(FI1):f63‐f71. doi:10.1093/ehjcvp/pvaa039 32339234

[45] Xu W , Huang X , Lin Q , et al. Application of alfalfa app in the management of oral anticoagulation in patients with atrial fibrillation: a multicenter randomized controlled trial. BMC Med Inform Decis Mak. 2024;24(1):294. doi:10.1186/s12911-024-02701-1 PMC11465833 39385171

[46] Desteghe L , Delesie M , Knaepen L , et al. Effect of targeted education of patients with atrial fibrillation on unplanned cardiovascular outcomes: results of the multicentre randomized AF‐EduCare trial. Europace. 2025;27(1):euae211. doi:10.1093/europace/euae211 PMC11745127 39832209

[47] Kunneman M , Branda ME , Noseworthy PA , et al. Shared decision making for stroke prevention in atrial fibrillation: study protocol for a randomized controlled trial. Trials. 2017;18(1):443. doi:10.1186/s13063-017-2178-y PMC5622521 28962662

[48] Al‐Arkee S , Mason J , Lane DA , et al. Mobile apps to improve medication adherence in cardiovascular disease: systematic review and meta‐analysis. J Med Internet Res. 2021;23(5):e24190. doi:10.2196/24190 PMC8188316 34032583

[49] Santo K , Singleton A , Rogers K , et al. Medication reminder applications to improve adherence in coronary heart disease: a randomised clinical trial. Heart. 2019;105(4):323‐329. doi:10.1136/heartjnl-2018-313479 30150326

[50] Sarfo FS , Treiber F , Gebregziabher M , et al. Phone‐based intervention for blood pressure control among Ghanaian stroke survivors: a pilot randomized controlled trial. Int J Stroke. 2019;14(6):630‐638. doi:10.1177/1747493018816423 30465630

[51] Bond Z , Scanlon T , Judah G . Systematic review of RCTs assessing the effectiveness of mHealth interventions to improve statin medication adherence: using the behaviour‐change technique taxonomy to identify the techniques that improve adherence. Healthcare (Basel). 2021;9(10):1282. doi:10.3390/healthcare9101282 PMC8535703 34682962

[52] Anderson LJ , Nuckols TK , Coles C , et al. A systematic overview of systematic reviews evaluating medication adherence interventions. Am J Health Syst Pharm. 2020;77(2):138‐147. doi:10.1093/ajhp/zxz284 PMC6946938 31901098

[53] Jang I . A systematic review on mobile health applications' education program for patients taking oral anticoagulants. Int J Environ Res Public Health. 2021;18(17):8902. doi:10.3390/ijerph18178902 PMC8430962 34501492

[54] Ingason AB , Hreinsson JP , Lund SH , et al. Comparison of medication adherence to different oral anticoagulants: population‐based cohort study. BMJ Open. 2023;13(1):e065700. doi:10.1136/bmjopen-2022-065700 PMC9827257 36604126

[55] Wilder ME , Kulie P , Jensen C , et al. The impact of social determinants of health on medication adherence: a systematic review and meta‐analysis. J Gen Intern Med. 2021;36(5):1359‐1370. doi:10.1007/s11606-020-06447-0 PMC8131473 33515188

[56] Salmasi S , Loewen PS , Tandun R , Andrade JG , De Vera MA . Adherence to oral anticoagulants among patients with atrial fibrillation: a systematic review and meta‐analysis of observational studies. BMJ Open. 2020;10(4):e034778. doi:10.1136/bmjopen-2019-034778 PMC7245382 32273316

[57] Alberts MJ , Peacock WF , Fields LE , et al. Association between once‐ and twice‐daily direct oral anticoagulant adherence in nonvalvular atrial fibrillation patients and rates of ischemic stroke. Int J Cardiol. 2016;215:11‐113. doi:10.1016/j.ijcard.2016.03.212 27104919

[58] Deshpande CG , Kogut S , Laforge R , Willey C . Impact of medication adherence on risk of ischemic stroke, major bleeding and deep vein thrombosis in atrial fibrillation patients using novel oral anticoagulants. Curr Med Res Opin. 2018;34(7):1285‐1292. doi:10.1080/03007995.2018.1428543 29334815

[59] Safari A , Helisaz H , Salmasi S , et al. Association between oral anticoagulant adherence and serious clinical outcomes in patients with atrial fibrillation: a long‐term retrospective cohort study. JAHA. 2024;13(18):e035639. doi:10.1161/JAHA.124.035639 PMC11935623 39248280

[60] Borne RT , O'Donnell C , Turakhia MP , et al. Adherence and outcomes to direct oral anticoagulants among patients with atrial fibrillation: findings from the veterans health administration. BMC Cardiovasc Disord. 2017;17(1):236. doi:10.1186/s12872-017-0671-6 PMC5581418 28865440

[61] Shore S , Carey EP , Turakhia MP , et al. Adherence to dabigatran therapy and longitudinal patient outcomes: insights from the veterans health administration. Am Heart J. 2014;167(6):810‐817. doi:10.1016/j.ahj.2014.03.023 PMC5381802 24890529

[62] Yao X , Abraham NS , Alexander GC , et al. Effect of adherence to oral anticoagulants on risk of stroke and major bleeding among patients with atrial fibrillation. J Am Heart Assoc. 2016;5(2):e003074. doi:10.1161/JAHA.115.003074 PMC4802483 26908412

