# Supplementary material for: Mobile health interventions to improve adherence to oral anticoagulant treatment: A systematic review and critical analysis
Source: Br J Clin Pharmacol. 2026 Jul 31;92(11):3733–58. doi: 10.1002/bcp.70718 (PMC13714613; doi:10.1002/bcp.70718)
Supplement: Supplementary file 1 [file BCP-92-3733-s001.docx]

Appendix 1

| Database | Search strategy |
| --- | --- |
| PubMed | 1. Mobile application* 2. Mobile health 3. mHealth 4. ehealth 5. Telemedicine 6. Messag* 7. Smart phone* 8. Smartphone* 9. Cell phone* 10. Mobile phone* 11. Home monitoring 12. Technolog* 13. Digital health 14. App 15. Application* 16. Wearable* 17. Smart watch 18. Smart device 19. Electronic 20. Reminder* 21. 1 OR 2 OR 3 OR 4 OR 5 OR 6 OR 7 OR 8 OR 9 OR 10 OR 11 OR 12 OR 13 OR 14 OR 15 OR 16 OR 17 OR 18 OR 19 OR 20 22. Oral anticoagul* 23. Anticoagul* 24. Dabigatran 25. Rivaroxaban 26. Apixaban 27. Edoxaban 28. Betrixaban 29. Warfarin 30. 22 OR 23 OR 24 OR 25 OR 26 OR 27 28 OR 29 31. Medication adherence 32. Adhere* 33. Complian* 34. Persistence 35. Non adherence 36. Non compliance 37. Non persistence 38. Complier* 39. 31 OR 32 OR 33 OR 34 OR 35 OR 36 OR 37 OR 38 40. Randomized controlled trial 41. Randomised controlled trial 42. Controlled clinical trial 43. Randomized 44. Randomised 45. Randomization 46. Randomisation 47. 40 OR 41 OR 42 OR 43 OR 44 OR 46 48. 21 AND 30 AND 39 AND 47 |
| Embase | 1. 'mobile application*' 2. 'mobile health' 3. mHealth 4. ehealth 5. telemedicine 6. messag* 7. 'smart phone*' 8. smartphone* 9. 'cell phone*' 10. 'mobile phone*' 11. 'home monitoring' 12. technolog* 13. 'digital health' 14. app 15. application* 16. wearable* 17. 'smart watch' 18. 'smart device' 19. electronic 20. reminder* 21. 1 OR 2 OR 3 OR 4 OR 5 OR 6 OR 7 OR 8 OR 9 OR 10 OR 11 OR 12 OR 13 OR 14 OR 15 OR 16 OR 17 OR 18 OR 19 OR 20 22. 'oral anticoagul*' 23. anticoagul* 24. dabigatran 25. rivaroxaban 26. apixaban 27. edoxaban 28. betrixaban 29. warfarin 30. 22 OR 23 OR 24 OR 25 OR 26 OR 27 OR 28 OR 29 31. 'medication adherence' 32. adhere* 33. complian* 34. persistence 35. 'non adherence' 36. 'non compliance' 37. 'non persistence' 38. Complier* 39. 31 OR 32 OR 33 OR 34 OR 35 OR 36 OR 37 OR 38 40. 'randomized controlled trial' 41. 'randomised controlled trial' 42. 'controlled clinical trial' 43. randomized 44. randomised 45. randomization 46. randomisation 47. 40 OR 41 OR 42 OR 43 OR 44 OR 45 OR 46 48. 21 AND 30 AND 39 AND 47 |
| Web of Science | 1. ALL=(Mobile application*) 2. ALL=(Mobile health) 3. ALL=(mHealth) 4. ALL=(ehealth) 5. ALL=(Telemedicine) 6. ALL=(messag*) 7. ALL=(Smart phone*) 8. ALL=(Smartphone*) 9. ALL=(Cell phone*) 10. ALL=(Mobile phone*) 11. ALL=(Home monitoring) 12. ALL=(Technolog*) 13. ALL=(Digital health) 14. ALL=(App) 15. ALL=(Application*) 16. ALL=(Wearable*) 17. ALL=(Smart watch) 18. ALL=(Smart device) 19. ALL=(Electronic) 20. ALL=(Reminder*) 21. 1 OR 2 OR 3 OR 4 OR 5 OR 6 OR 7 OR 8 OR 9 OR 10 OR 11 OR 12 OR 13 OR 14 OR 15 OR 16 OR 17 OR 18 OR 19 OR 20 22. ALL=(Oral anticoagul*) 23. ALL=(Anticoagul*) 24. ALL=(Dabigatran) 25. ALL=(Rivaroxaban) 26. ALL=(Apixaban) 27. ALL=(Edoxaban) 28. ALL=(Betrixaban) 29. ALL=(Warfarin) 30. 22 OR 23 OR 24 OR 25 OR 26 OR 27 28 OR 29 31. ALL=(Medication adherence) 32. ALL=(Adhere*) 33. ALL=( Complian*) 34. ALL=(Persistence) 35. ALL=(Non adherence) 36. ALL=(Non compliance) 37. ALL=(Non persistence) 38. ALL=(Complier*) 39. 31 OR 32 OR 33 OR 34 OR 35 OR 36 OR 37 OR 38 40. ALL=(Randomized controlled trial) 41. ALL=(Randomised controlled trial) 42. ALL=(Controlled clinical trial) 43. ALL=(Randomized) 44. ALL=(Randomised) 45. ALL=(Randomization) 46. ALL=(Randomisation) 47. 40 OR 41 OR 42 OR 43 OR 44 OR 45 OR 46 48. 21 AND 30 AND 39 AND 47 |
| Cochrane | 1. (“mobile application*”) 2. (“mobile health”) 3. (mHealth) 4. (ehealth) 5. (“telemedicine”) 6. (messag*) 7. (“smart phone*”) 8. (smartphone*) 9. (“cell phone*”) 10. (“mobile phone*”) 11. (“home monitoring”) 12. (technolog*) 13. (“digital health”) 14. (app) 15. (application*) 16. (wearable*) 17. (“smart watch”) 18. (“smart device”) 19. (electronic) 20. (reminder*) 21. 1 OR 2 OR 3 OR 4 OR 5 OR 6 OR 7 OR 8 OR 9 OR 10 OR 11 OR 12 OR 13 OR 14 OR 15 OR 16 OR 17 OR 18 OR 19 OR 20 22. (“oral anticoagul*”) 23. (anticoagul*) 24. (dabigatran) 25. (rivaroxaban) 26. (apixaban) 27. (edoxaban) 28. (betrixaban) 29. (warfarin) 30. 22 OR 23 OR 24 OR 25 OR 26 OR 27 OR 28 OR 29 31. (“medication adherence”) 32. (adhere*) 33. (complian*) 34. (persistence) 35. (“non adherence”) 36. (“non compliance”) 37. (“non persistence”) 38. (complier*) 39. 31 OR 32 OR 33 OR 34 OR 35 OR 36 OR 37 OR 38 40. (“randomized controlled trial”) 41. (“randomised controlled trial”) 42. (“controlled clinical trial”) 43. (randomised) 44. (randomized) 45. (randomization) 46. (randomisation) 47. 40 OR 41 OR 42 OR 43 OR 44 OR 45 OR 46 48. 21 AND 30 AND 39 AND 47 |
